# Supplementary material for: Traumatic Brain Injury Characteristics Predictive of Subsequent Sleep-Wake Disturbances in Pediatric Patients
Source: Biology (Basel). 2022 Apr 14;11(4):600. doi: 10.3390/biology11040600 (PMC9030185; doi:10.3390/biology11040600)
Supplement: Supplementary file 1 [file biology-11-00600-s001.zip › biology-1652474-supplementary.pdf]

Supplementary Table S1. ICD 9 and ICD 10 codes for sleep-wake disturbances (SWD) and traumatic brain injury (TBI).

ICD 9 codes SWD

32700	Organic insomnia, unspecified
32701	Insomnia due to medical condition classified elsewhere
32702	Insomnia due to mental disorder
32709	Other organic insomnia
32710	Organic hypersomnia, unspecified
32711	Idiopathic hypersomnia with long sleep time
32712	Idiopathic hypersomnia without long sleep time
32713	Recurrent hypersomnia
32714	Hypersomnia due to medical condition classified elsewhere
32715	Hypersomnia due to mental disorder
32719	Other organic hypersomnia
32720	Organic sleep apnea, unspecified
32721	Primary central sleep apnea
32722	High altitude periodic breathing
32723	Obstructive sleep apnea (adult)(pediatric)
32724	Idiopathic sleep related non-obstructive alveolar hypoventilation
32725	Congenital central alveolar hypoventilation syndrome
32726	Sleep related hypoventilation/hypoxemia in conditions classifiable elsewhere
32727	Central sleep apnea in conditions classified elsewhere
32729	Other organic sleep apnea
32730	Circadian rhythm sleep disorder, unspecified
32731	Circadian rhythm sleep disorder, delayed sleep phase type
32732	Circadian rhythm sleep disorder, advanced sleep phase type
32733	Circadian rhythm sleep disorder, irregular sleep-wake type
32734	Circadian rhythm sleep disorder, free-running type
32735	Circadian rhythm sleep disorder, jet lag type
32736	Circadian rhythm sleep disorder, shift work type
32737	Circadian rhythm sleep disorder in conditions classified elsewhere
32739	Other circadian rhythm sleep disorder
32740	Organic parasomnia, unspecified
32741	Confusional arousals
32742	REM sleep behavior disorder
32743	Recurrent isolated sleep paralysis
32744	Parasomnia in conditions classified elsewhere
32749	Other organic parasomnia
32751	Periodic limb movement disorder
32752	Sleep related leg cramps

32753	Sleep related bruxism
32759	Other organic sleep related movement disorders
3278	Other organic sleep disorders
34700	Narcolepsy, without cataplexy
34701	Narcolepsy, with cataplexy
34710	Narcolepsy in conditions classified elsewhere, without cataplexy
34711	Narcolepsy in conditions classified elsewhere, with cataplexy
78050	Sleep disturbance, unspecified
78051	Insomnia with sleep apnea, unspecified
78052	Insomnia, unspecified
78053	Hypersomnia with sleep apnea, unspecified
78054	Hypersomnia, unspecified
78055	Disruption of 24 hour sleep wake cycle, unspecified
78056	Dysfunctions associated with sleep stages or arousal from sleep
78057	Unspecified sleep apnea
78058	Sleep related movement disorder, unspecified
78059	Other sleep disturbances

ICD 9 Codes TBI

33920	Post-traumatic headache, unspecified
33921	Acute post-traumatic headache
33922	Chronic post-traumatic headache
3484	Compression of brain
3485	Cerebral edema
78001	Coma
80002	Closed fracture of vault of skull without mention of intracranial injury, with brief [less than one hour] loss of consciousness
80003	Closed fracture of vault of skull without mention of intracranial injury, with moderate [1-24 hours] loss of consciousness
80004	Closed fracture of vault of skull without mention of intracranial injury, with prolonged [more than 24 hours] loss of consciousness and return to pre-existing conscious level
80005	Closed fracture of vault of skull without mention of intracranial injury, with prolonged [more than 24 hours] loss of consciousness, without return to pre-existing conscious level
80006	Closed fracture of vault of skull without mention of intracranial injury, with loss of consciousness of unspecified duration
80009	Closed fracture of vault of skull without mention of intracranial injury, with concussion, unspecified
80010	Closed fracture of vault of skull with cerebral laceration and contusion, unspecified state of consciousness
80011	Closed fracture of vault of skull with cerebral laceration and contusion, with no loss of consciousness

80012	Closed fracture of vault of skull with cerebral laceration and contusion, with brief [less than one hour] loss of consciousness
80013	Closed fracture of vault of skull with cerebral laceration and contusion, with moderate [1-24 hours] loss of consciousness
80014	Closed fracture of vault of skull with cerebral laceration and contusion, with prolonged [more than 24 hours] loss of consciousness and return to pre-existing conscious level
80015	Closed fracture of vault of skull with cerebral laceration and contusion, with prolonged [more than 24 hours] loss of consciousness, without return to pre-existing conscious level
80016	Closed fracture of vault of skull with cerebral laceration and contusion, with loss of consciousness of unspecified duration
80019	Closed fracture of vault of skull with cerebral laceration and contusion, with concussion, unspecified
80020	Closed fracture of vault of skull with subarachnoid, subdural, and extradural hemorrhage, unspecified state of consciousness
80021	Closed fracture of vault of skull with subarachnoid, subdural, and extradural hemorrhage, with no loss of consciousness
80022	Closed fracture of vault of skull with subarachnoid, subdural, and extradural hemorrhage, with brief [less than one hour] loss of consciousness
80023	Closed fracture of vault of skull with subarachnoid, subdural, and extradural hemorrhage, with moderate [1-24 hours] loss of consciousness
80024	Closed fracture of vault of skull with subarachnoid, subdural, and extradural hemorrhage, with prolonged [more than 24 hours] loss of consciousness and return to pre-existing conscious level
80025	Closed fracture of vault of skull with subarachnoid, subdural, and extradural hemorrhage, with prolonged [more than 24 hours] loss of consciousness, without return to pre-existing conscious level
80026	Closed fracture of vault of skull with subarachnoid, subdural, and extradural hemorrhage, with loss of consciousness of unspecified duration
80029	Closed fracture of vault of skull with subarachnoid, subdural, and extradural hemorrhage, with concussion, unspecified
80030	Closed fracture of vault of skull with other and unspecified intracranial hemorrhage, unspecified state of consciousness
80031	Closed fracture of vault of skull with other and unspecified intracranial hemorrhage, with no loss of consciousness
80032	Closed fracture of vault of skull with other and unspecified intracranial hemorrhage, with brief [less than one hour] loss of consciousness
80033	Closed fracture of vault of skull with other and unspecified intracranial hemorrhage, with moderate [1-24 hours] loss of consciousness
80034	Closed fracture of vault of skull with other and unspecified intracranial hemorrhage, with prolonged [more than 24 hours] loss of consciousness and return to pre-existing conscious level
80035	Closed fracture of vault of skull with other and unspecified intracranial hemorrhage, with prolonged [more than 24 hours] loss of consciousness, without return to pre-existing conscious level
80036	Closed fracture of vault of skull with other and unspecified intracranial hemorrhage, with loss of consciousness of unspecified duration

80039	Closed fracture of vault of skull with other and unspecified intracranial hemorrhage, with concussion, unspecified
80040	Closed fracture of vault of skull with intracranial injury of other and unspecified nature, unspecified state of consciousness
80041	Closed fracture of vault of skull with intracranial injury of other and unspecified nature, with no loss of consciousness
80042	Closed fracture of vault of skull with intracranial injury of other and unspecified nature, with brief [less than one hour] loss of consciousness
80043	Closed fracture of vault of skull with intracranial injury of other and unspecified nature, with moderate [1-24 hours] loss of consciousness
80044	Closed fracture of vault of skull with intracranial injury of other and unspecified nature, with prolonged [more than 24 hours] loss of consciousness and return to pre-existing conscious level
80045	Closed fracture of vault of skull with intracranial injury of other and unspecified nature, with prolonged [more than 24 hours] loss of consciousness, without return to pre-existing conscious level
80046	Closed fracture of vault of skull with intracranial injury of other and unspecified nature, with loss of consciousness of unspecified duration
80049	Closed fracture of vault of skull with intracranial injury of other and unspecified nature, with concussion, unspecified
80050	Open fracture of vault of skull without mention of intracranial injury, unspecified state of consciousness
80051	Open fracture of vault of skull without mention of intracranial injury, with no loss of consciousness
80052	Open fracture of vault of skull without mention of intracranial injury, with brief [less than one hour] loss of consciousness
80053	Open fracture of vault of skull without mention of intracranial injury, with moderate [1-24 hours] loss of consciousness
80054	Open fracture of vault of skull without mention of intracranial injury, with prolonged [more than 24 hours] loss of consciousness and return to pre-existing conscious level
80055	Open fracture of vault of skull without mention of intracranial injury, with prolonged [more than 24 hours] loss of consciousness, without return to pre-existing conscious level
80056	Open fracture of vault of skull without mention of intracranial injury, with loss of consciousness of unspecified duration
80059	Open fracture of vault of skull without mention of intracranial injury, with concussion, unspecified
80060	Open fracture of vault of skull with cerebral laceration and contusion, unspecified state of consciousness
80061	Open fracture of vault of skull with cerebral laceration and contusion, with no loss of consciousness
80062	Open fracture of vault of skull with cerebral laceration and contusion, with brief [less than one hour] loss of consciousness
80063	Open fracture of vault of skull with cerebral laceration and contusion, with moderate [1-24 hours] loss of consciousness
80064	Open fracture of vault of skull with cerebral laceration and contusion, with prolonged [more than 24 hours] loss of consciousness and return to pre-existing conscious level

80065	Open fracture of vault of skull with cerebral laceration and contusion, with prolonged [more than 24 hours] loss of consciousness, without return to pre-existing conscious level
80066	Open fracture of vault of skull with cerebral laceration and contusion, with loss of consciousness of unspecified duration
80069	Open fracture of vault of skull with cerebral laceration and contusion, with concussion, unspecified
80070	Open fracture of vault of skull with subarachnoid, subdural, and extradural hemorrhage, unspecified state of consciousness
80071	Open fracture of vault of skull with subarachnoid, subdural, and extradural hemorrhage, with no loss of consciousness
80072	Open fracture of vault of skull with subarachnoid, subdural, and extradural hemorrhage, with brief [less than one hour] loss of consciousness
80073	Open fracture of vault of skull with subarachnoid, subdural, and extradural hemorrhage, with moderate [1-24 hours] loss of consciousness
80074	Open fracture of vault of skull with subarachnoid, subdural, and extradural hemorrhage, with prolonged [more than 24 hours] loss of consciousness and return to pre-existing conscious level
80075	Open fracture of vault of skull with subarachnoid, subdural, and extradural hemorrhage, with prolonged [more than 24 hours] loss of consciousness, without return to pre-existing conscious level
80076	Open fracture of vault of skull with subarachnoid, subdural, and extradural hemorrhage, with loss of consciousness of unspecified duration
80079	Open fracture of vault of skull with subarachnoid, subdural, and extradural hemorrhage, with concussion, unspecified
80080	Open fracture of vault of skull with other and unspecified intracranial hemorrhage, unspecified state of consciousness
80081	Open fracture of vault of skull with other and unspecified intracranial hemorrhage, with no loss of consciousness
80082	Open fracture of vault of skull with other and unspecified intracranial hemorrhage, with brief [less than one hour] loss of consciousness
80083	Open fracture of vault of skull with other and unspecified intracranial hemorrhage, with moderate [1-24 hours] loss of consciousness
80084	Open fracture of vault of skull with other and unspecified intracranial hemorrhage, with prolonged [more than 24 hours] loss of consciousness and return to pre-existing conscious level
80085	Open fracture of vault of skull with other and unspecified intracranial hemorrhage, with prolonged [more than 24 hours] loss of consciousness, without return to pre-existing conscious level
80086	Open fracture of vault of skull with other and unspecified intracranial hemorrhage, with loss of consciousness of unspecified duration
80089	Open fracture of vault of skull with other and unspecified intracranial hemorrhage, with concussion, unspecified
80090	Open fracture of vault of skull with intracranial injury of other and unspecified nature, unspecified state of consciousness
80091	Open fracture of vault of skull with intracranial injury of other and unspecified nature, with no loss of consciousness

80092	Open fracture of vault of skull with intracranial injury of other and unspecified nature, with brief [less than one hour] loss of consciousness
80093	Open fracture of vault of skull with intracranial injury of other and unspecified nature, with moderate [1-24 hours] loss of consciousness
80094	Open fracture of vault of skull with intracranial injury of other and unspecified nature, with prolonged [more than 24 hours] loss of consciousness and return to pre-existing conscious level
80095	Open fracture of vault of skull with intracranial injury of other and unspecified nature, with prolonged [more than 24 hours] loss of consciousness, without return to pre-existing conscious level
80096	Open fracture of vault of skull with intracranial injury of other and unspecified nature, with loss of consciousness of unspecified duration
80099	Open fracture of vault of skull with intracranial injury of other and unspecified nature, with concussion, unspecified
80100	Closed fracture of base of skull without mention of intra cranial injury, unspecified state of consciousness
80101	Closed fracture of base of skull without mention of intra cranial injury, with no loss of consciousness
80102	Closed fracture of base of skull without mention of intra cranial injury, with brief [less than one hour] loss of consciousness
80103	Closed fracture of base of skull without mention of intra cranial injury, with moderate [1-24 hours] loss of consciousness
80104	Closed fracture of base of skull without mention of intra cranial injury, with prolonged [more than 24 hours] loss of consciousness and return to pre-existing conscious level
80105	Closed fracture of base of skull without mention of intra cranial injury, with prolonged [more than 24 hours] loss of consciousness, without return to pre-existing conscious level
80106	Closed fracture of base of skull without mention of intra cranial injury, with loss of consciousness of unspecified duration
80109	Closed fracture of base of skull without mention of intra cranial injury, with concussion, unspecified
80110	Closed fracture of base of skull with cerebral laceration and contusion, unspecified state of consciousness
80111	Closed fracture of base of skull with cerebral laceration and contusion, with no loss of consciousness
80112	Closed fracture of base of skull with cerebral laceration and contusion, with brief [less than one hour] loss of consciousness
80113	Closed fracture of base of skull with cerebral laceration and contusion, with moderate [1-24 hours] loss of consciousness
80114	Closed fracture of base of skull with cerebral laceration and contusion, with prolonged [more than 24 hours] loss of consciousness and return to pre-existing conscious level
80115	Closed fracture of base of skull with cerebral laceration and contusion, with prolonged [more than 24 hours] loss of consciousness, without return to pre-existing conscious level
80116	Closed fracture of base of skull with cerebral laceration and contusion, with loss of consciousness of unspecified duration
80119	Closed fracture of base of skull with cerebral laceration and contusion, with concussion, unspecified

80120	Closed fracture of base of skull with subarachnoid, subdural, and extradural hemorrhage, unspecified state of consciousness
80121	Closed fracture of base of skull with subarachnoid, subdural, and extradural hemorrhage, with no loss of consciousness
80122	Closed fracture of base of skull with subarachnoid, subdural, and extradural hemorrhage, with brief [less than one hour] loss of consciousness
80123	Closed fracture of base of skull with subarachnoid, subdural, and extradural hemorrhage, with moderate [1-24 hours] loss of consciousness
80124	Closed fracture of base of skull with subarachnoid, subdural, and extradural hemorrhage, with prolonged [more than 24 hours] loss of consciousness and return to pre-existing conscious level
80125	Closed fracture of base of skull with subarachnoid, subdural, and extradural hemorrhage, with prolonged [more than 24 hours] loss of consciousness, without return to pre-existing conscious level
80126	Closed fracture of base of skull with subarachnoid, subdural, and extradural hemorrhage, with loss of consciousness of unspecified duration
80129	Closed fracture of base of skull with subarachnoid, subdural, and extradural hemorrhage, with concussion, unspecified
80130	Closed fracture of base of skull with other and unspecified intracranial hemorrhage, unspecified state of consciousness
80131	Closed fracture of base of skull with other and unspecified intracranial hemorrhage, with no loss of consciousness
80132	Closed fracture of base of skull with other and unspecified intracranial hemorrhage, with brief [less than one hour] loss of consciousness
80133	Closed fracture of base of skull with other and unspecified intracranial hemorrhage, with moderate [1-24 hours] loss of consciousness
80134	Closed fracture of base of skull with other and unspecified intracranial hemorrhage, with prolonged [more than 24 hours] loss of consciousness and return to pre-existing conscious level
80135	Closed fracture of base of skull with other and unspecified intracranial hemorrhage, with prolonged [more than 24 hours] loss of consciousness, without return to pre-existing conscious level
80136	Closed fracture of base of skull with other and unspecified intracranial hemorrhage, with loss of consciousness of unspecified duration
80139	Closed fracture of base of skull with other and unspecified intracranial hemorrhage, with concussion, unspecified
80140	Closed fracture of base of skull with intracranial injury of other and unspecified nature, unspecified state of consciousness
80141	Closed fracture of base of skull with intracranial injury of other and unspecified nature, with no loss of consciousness
80142	Closed fracture of base of skull with intracranial injury of other and unspecified nature, with brief [less than one hour] loss of consciousness
80143	Closed fracture of base of skull with intracranial injury of other and unspecified nature, with moderate [1-24 hours] loss of consciousness

80144	Closed fracture of base of skull with intracranial injury of other and unspecified nature, with prolonged [more than 24 hours) loss of consciousness and return to pre-existing conscious level
80145	Closed fracture of base of skull with intracranial injury of other and unspecified nature, with prolonged [more than 24 hours] loss of consciousness, without return to pre-existing conscious level
80146	Closed fracture of base of skull with intracranial injury of other and unspecified nature, with loss of consciousness of unspecified duration
80149	Closed fracture of base of skull with intracranial injury of other and unspecified nature, with concussion, unspecified
80150	Open fracture of base of skull without mention of intracranial injury, unspecified state of consciousness
80151	Open fracture of base of skull without mention of intracranial injury, with no loss of consciousness
80152	Open fracture of base of skull without mention of intracranial injury, with brief [less than one hour] loss of consciousness
80153	Open fracture of base of skull without mention of intracranial injury, with moderate [1-24 hours] loss of consciousness
80154	Open fracture of base of skull without mention of intracranial injury, with prolonged [more than 24 hours] loss of consciousness and return to pre-existing conscious level
80155	Open fracture of base of skull without mention of intracranial injury, with prolonged [more than 24 hours] loss of consciousness, without return to pre-existing conscious level
80156	Open fracture of base of skull without mention of intracranial injury, with loss of consciousness of unspecified duration
80159	Open fracture of base of skull without mention of intracranial injury, with concussion, unspecified
80160	Open fracture of base of skull with cerebral laceration and contusion, unspecified state of consciousness
80161	Open fracture of base of skull with cerebral laceration and contusion, with no loss of consciousness
80162	Open fracture of base of skull with cerebral laceration and contusion, with brief [less than one hour] loss of consciousness
80163	Open fracture of base of skull with cerebral laceration and contusion, with moderate [1-24 hours] loss of consciousness
80164	Open fracture of base of skull with cerebral laceration and contusion, with prolonged [more than 24 hours] loss of consciousness and return to pre-existing conscious level
80165	Open fracture of base of skull with cerebral laceration and contusion, with prolonged [more than 24 hours] loss of consciousness, without return to pre-existing conscious level
80166	Open fracture of base of skull with cerebral laceration and contusion, with loss of consciousness of unspecified duration
80169	Open fracture of base of skull with cerebral laceration and contusion, with concussion, unspecified
80170	Open fracture of base of skull with subarachnoid, subdural, and extradural hemorrhage, unspecified state of consciousness
80171	Open fracture of base of skull with subarachnoid, subdural, and extradural hemorrhage, with no loss of consciousness

80172	Open fracture of base of skull with subarachnoid, subdural, and extradural hemorrhage, with brief [less than one hour] loss of consciousness
80173	Open fracture of base of skull with subarachnoid, subdural, and extradural hemorrhage, with moderate [1-24 hours] loss of consciousness
80174	Open fracture of base of skull with subarachnoid, subdural, and extradural hemorrhage, with prolonged [more than 24 hours] loss of consciousness and return to pre-existing conscious level
80175	Open fracture of base of skull with subarachnoid, subdural, and extradural hemorrhage, with prolonged [more than 24 hours] loss of consciousness, without return to pre-existing conscious level
80176	Open fracture of base of skull with subarachnoid, subdural, and extradural hemorrhage, with loss of consciousness of unspecified duration
80179	Open fracture of base of skull with subarachnoid, subdural, and extradural hemorrhage, with concussion, unspecified
80180	Open fracture of base of skull with other and unspecified intracranial hemorrhage, unspecified state of consciousness
80181	Open fracture of base of skull with other and unspecified intracranial hemorrhage, with no loss of consciousness
80182	Open fracture of base of skull with other and unspecified intracranial hemorrhage, with brief [less than one hour] loss of consciousness
80183	Open fracture of base of skull with other and unspecified intracranial hemorrhage, with moderate [1-24 hours] loss of consciousness
80184	Open fracture of base of skull with other and unspecified intracranial hemorrhage, with prolonged [more than 24 hours] loss of consciousness and return to pre-existing conscious level
80185	Open fracture of base of skull with other and unspecified intracranial hemorrhage, with prolonged [more than 24 hours] loss of consciousness, without return to pre-existing conscious level
80186	Open fracture of base of skull with other and unspecified intracranial hemorrhage, with loss of consciousness of unspecified duration
80189	Open fracture of base of skull with other and unspecified intracranial hemorrhage, with concussion, unspecified
80190	Open fracture of base of skull with intracranial injury of other and unspecified nature, unspecified state of consciousness
80191	Open fracture of base of skull with intracranial injury of other and unspecified nature, with no loss of consciousness
80192	Open fracture of base of skull with intracranial injury of other and unspecified nature, with brief [less than one hour] loss of consciousness
80193	Open fracture of base of skull with intracranial injury of other and unspecified nature, with moderate [1-24 hours] loss of consciousness
80194	Open fracture of base of skull with intracranial injury of other and unspecified nature, with prolonged [more than 24 hours] loss of consciousness and return to pre-existing conscious level
80195	Open fracture of base of skull with intracranial injury of other and unspecified nature, with prolonged [more than 24 hours] loss of consciousness, without return to pre-existing conscious level

80196	Open fracture of base of skull with intracranial injury of other and unspecified nature, with loss of consciousness of unspecified duration
80199	Open fracture of base of skull with intracranial injury of other and unspecified nature, with concussion, unspecified
80300	Other closed skull fracture without mention of intracranial injury, unspecified state of consciousness
80301	Other closed skull fracture without mention of intracranial injury, with no loss of consciousness
80302	Other closed skull fracture without mention of intracranial injury, with brief [less than one hour] loss of consciousness
80303	Other closed skull fracture without mention of intracranial injury, with moderate [1-24 hours] loss of consciousness
80304	Other closed skull fracture without mention of intracranial injury, with prolonged [more than 24 hours] loss of consciousness and return to pre-existing conscious level
80305	Other closed skull fracture without mention of intracranial injury, with prolonged [more than 24 hours] loss of consciousness, without return to pre-existing conscious level
80306	Other closed skull fracture without mention of intracranial injury, with loss of consciousness of unspecified duration
80309	Other closed skull fracture without mention of intracranial injury, with concussion, unspecified
80310	Other closed skull fracture with cerebral laceration and contusion, unspecified state of consciousness
80311	Other closed skull fracture with cerebral laceration and contusion, with no loss of consciousness
80312	Other closed skull fracture with cerebral laceration and contusion, with brief [less than one hour] loss of consciousness
80313	Other closed skull fracture with cerebral laceration and contusion, with moderate [1-24 hours] loss of consciousness
80314	Other closed skull fracture with cerebral laceration and contusion, with prolonged [more than 24 hours] loss of consciousness and return to pre-existing conscious level
80315	Other closed skull fracture with cerebral laceration and contusion, with prolonged [more than 24 hours] loss of consciousness, without return to pre-existing conscious level
80316	Other closed skull fracture with cerebral laceration and contusion, with loss of consciousness of unspecified duration
80319	Other closed skull fracture with cerebral laceration and contusion, with concussion, unspecified
80320	Other closed skull fracture with subarachnoid, subdural, and extradural hemorrhage, unspecified state of consciousness
80321	Other closed skull fracture with subarachnoid, subdural, and extradural hemorrhage, with no loss of consciousness
80322	Other closed skull fracture with subarachnoid, subdural, and extradural hemorrhage, with brief [less than one hour] loss of consciousness
80323	Other closed skull fracture with subarachnoid, subdural, and extradural hemorrhage, with moderate [1-24 hours] loss of consciousness

80324	Other closed skull fracture with subarachnoid, subdural, and extradural hemorrhage, with prolonged [more than 24 hours] loss of consciousness and return to pre-existing conscious level
80325	Other closed skull fracture with subarachnoid, subdural, and extradural hemorrhage, with prolonged [more than 24 hours] loss of consciousness, without return to pre-existing conscious level
80326	Other closed skull fracture with subarachnoid, subdural, and extradural hemorrhage, with loss of consciousness of unspecified duration
80329	Other closed skull fracture with subarachnoid, subdural, and extradural hemorrhage, with concussion, unspecified
80330	Other closed skull fracture with other and unspecified intracranial hemorrhage, unspecified state of unconsciousness
80331	Other closed skull fracture with other and unspecified intracranial hemorrhage, with no loss of consciousness
80332	Other closed skull fracture with other and unspecified intracranial hemorrhage, with brief [less than one hour] loss of consciousness
80333	Other closed skull fracture with other and unspecified intracranial hemorrhage, with moderate [1-24 hours] loss of consciousness
80334	Other closed skull fracture with other and unspecified intracranial hemorrhage, with prolonged [more than 24 hours] loss of consciousness and return to pre-existing conscious level
80335	Other closed skull fracture with other and unspecified intracranial hemorrhage, with prolonged [more than 24 hours] loss of consciousness, without return to pre-existing conscious level
80336	Other closed skull fracture with other and unspecified intracranial hemorrhage, with loss of consciousness of unspecified duration
80339	Other closed skull fracture with other and unspecified intracranial hemorrhage, with concussion, unspecified
80340	Other closed skull fracture with intracranial injury of other and unspecified nature, unspecified state of consciousness
80341	Other closed skull fracture with intracranial injury of other and unspecified nature, with no loss of consciousness
80342	Other closed skull fracture with intracranial injury of other and unspecified nature, with brief [less than one hour] loss of consciousness
80343	Other closed skull fracture with intracranial injury of other and unspecified nature, with moderate [1-24 hours] loss of consciousness
80344	Other closed skull fracture with intracranial injury of other and unspecified nature, with prolonged [more than 24 hours] loss of consciousness and return to pre-existing conscious level
80345	Other closed skull fracture with intracranial injury of other and unspecified nature, with prolonged [more than 24 hours] loss of consciousness, without return to pre-existing conscious level
80346	Other closed skull fracture with intracranial injury of other and unspecified nature, with loss of consciousness of unspecified duration
80349	Other closed skull fracture with intracranial injury of other and unspecified nature, with concussion, unspecified

80350	Other open skull fracture without mention of injury, unspecified state of consciousness
80351	Other open skull fracture without mention of intracranial injury, with no loss of consciousness
80352	Other open skull fracture without mention of intracranial injury, with brief [less than one hour] loss of consciousness
80353	Other open skull fracture without mention of intracranial injury, with moderate [1-24 hours] loss of consciousness
80354	Other open skull fracture without mention of intracranial injury, with prolonged [more than 24 hours] loss of consciousness and return to pre-existing conscious level
80355	Other open skull fracture without mention of intracranial injury, with prolonged [more than 24 hours] loss of consciousness, without return to pre-existing conscious level
80356	Other open skull fracture without mention of intracranial injury, with loss of consciousness of unspecified duration
80359	Other open skull fracture without mention of intracranial injury, with concussion, unspecified
80360	Other open skull fracture with cerebral laceration and contusion, unspecified state of consciousness
80361	Other open skull fracture with cerebral laceration and contusion, with no loss of consciousness
80362	Other open skull fracture with cerebral laceration and contusion, with brief [less than one hour] loss of consciousness
80363	Other open skull fracture with cerebral laceration and contusion, with moderate [1-24 hours] loss of consciousness
80364	Other open skull fracture with cerebral laceration and contusion, with prolonged [more than 24 hours] loss of consciousness and return to pre-existing conscious level
80365	Other open skull fracture with cerebral laceration and contusion, with prolonged [more than 24 hours] loss of consciousness, without return to pre-existing conscious level
80366	Other open skull fracture with cerebral laceration and contusion, with loss of consciousness of unspecified duration
80369	Other open skull fracture with cerebral laceration and contusion, with concussion, unspecified
80370	Other open skull fracture with subarachnoid, subdural, and extradural hemorrhage, unspecified state of consciousness
80371	Other open skull fracture with subarachnoid, subdural, and extradural hemorrhage, with no loss of consciousness
80372	Other open skull fracture with subarachnoid, subdural, and extradural hemorrhage, with brief [less than one hour] loss of consciousness
80373	Other open skull fracture with subarachnoid, subdural, and extradural hemorrhage, with moderate [1-24 hours] loss of consciousness
80374	Other open skull fracture with subarachnoid, subdural, and extradural hemorrhage, with prolonged [more than 24 hours] loss of consciousness and return to pre-existing conscious level
80375	Other open skull fracture with subarachnoid, subdural, and extradural hemorrhage, with prolonged [more than 24 hours] loss of consciousness, without return to pre-existing conscious level

80376	Other open skull fracture with subarachnoid, subdural, and extradural hemorrhage, with loss of consciousness of unspecified duration
80379	Other open skull fracture with subarachnoid, subdural, and extradural hemorrhage, with concussion, unspecified
80380	Other open skull fracture with other and unspecified intracranial hemorrhage, unspecified state of consciousness
80381	Other open skull fracture with other and unspecified intracranial hemorrhage, with no loss of consciousness
80382	Other open skull fracture with other and unspecified intracranial hemorrhage, with brief [less than one hour] loss of consciousness
80383	Other open skull fracture with other and unspecified intracranial hemorrhage, with moderate [1-24 hours] loss of consciousness
80384	Other open skull fracture with other and unspecified intracranial hemorrhage, with prolonged [more than 24 hours] loss of consciousness and return to pre-existing conscious level
80385	Other open skull fracture with other and unspecified intracranial hemorrhage, with prolonged [more than 24 hours] loss of consciousness, without return to pre-existing conscious level
80386	Other open skull fracture with other and unspecified intracranial hemorrhage, with loss of consciousness of unspecified duration
80389	Other open skull fracture with other and unspecified intracranial hemorrhage, with concussion, unspecified
80390	Other open skull fracture with intracranial injury of other and unspecified nature, unspecified state of consciousness
80391	Other open skull fracture with intracranial injury of other and unspecified nature, with no loss of consciousness
80392	Other open skull fracture with intracranial injury of other and unspecified nature, with brief [less than one hour] loss of consciousness
80393	Other open skull fracture with intracranial injury of other and unspecified nature, with moderate [1-24 hours] loss of consciousness
80394	Other open skull fracture with intracranial injury of other and unspecified nature, with prolonged [more than 24 hours] loss of consciousness and return to pre-existing conscious level
80395	Other open skull fracture with intracranial injury of other and unspecified nature, with prolonged [more than 24 hours] loss of consciousness, without return to pre-existing conscious level
80396	Other open skull fracture with intracranial injury of other and unspecified nature, with loss of consciousness of unspecified duration
80399	Other open skull fracture with intracranial injury of other and unspecified nature, with concussion, unspecified
80400	Closed fractures involving skull or face with other bones, without mention of intracranial injury, unspecified state of consciousness
80401	Closed fractures involving skull or face with other bones, without mention of intracranial injury, with no loss of consciousness
80402	Closed fractures involving skull or face with other bones, without mention of intracranial injury, with brief [less than one hour] loss of consciousness

80403	Closed fractures involving skull or face with other bones, without mention of intracranial injury, with moderate [1-24 hours] loss of consciousness
80404	Closed fractures involving skull or face with other bones, without mention or intracranial injury, with prolonged [more than 24 hours] loss of consciousness and return to pre-existing conscious level
80405	Closed fractures involving skull of face with other bones, without mention of intracranial injury, with prolonged [more than 24 hours] loss of consciousness, without return to pre-existing conscious level
80406	Closed fractures involving skull of face with other bones, without mention of intracranial injury, with loss of consciousness of unspecified duration
80409	Closed fractures involving skull of face with other bones, without mention of intracranial injury, with concussion, unspecified
80410	Closed fractures involving skull or face with other bones, with cerebral laceration and contusion, unspecified state of consciousness
80411	Closed fractures involving skull or face with other bones, with cerebral laceration and contusion, with no loss of consciousness
80412	Closed fractures involving skull or face with other bones, with cerebral laceration and contusion, with brief [less than one hour] loss of consciousness
80413	Closed fractures involving skull or face with other bones, with cerebral laceration and contusion, with moderate [1-24 hours] loss of consciousness
80414	Closed fractures involving skull or face with other bones, with cerebral laceration and contusion, with prolonged [more than 24 hours] loss of consciousness and return to pre-existing conscious level
80415	Closed fractures involving skull or face with other bones, with cerebral laceration and contusion, with prolonged [more than 24 hours] loss of consciousness, without return to pre-existing conscious level
80416	Closed fractures involving skull or face with other bones, with cerebral laceration and contusion, with loss of consciousness of unspecified duration
80419	Closed fractures involving skull or face with other bones, with cerebral laceration and contusion, with concussion, unspecified
80420	Closed fractures involving skull or face with other bones with subarachnoid, subdural, and extradural hemorrhage, unspecified state of consciousness
80421	Closed fractures involving skull or face with other bones with subarachnoid, subdural, and extradural hemorrhage, with no loss of consciousness
80422	Closed fractures involving skull or face with other bones with subarachnoid, subdural, and extradural hemorrhage, with brief [less than one hour] loss of consciousness
80423	Closed fractures involving skull or face with other bones with subarachnoid, subdural, and extradural hemorrhage, with moderate [1-24 hours] loss of consciousness
80424	Closed fractures involving skull or face with other bones with subarachnoid, subdural, and extradural hemorrhage, with prolonged [more than 24 hours] loss of consciousness and return to pre-existing conscious level
80425	Closed fractures involving skull or face with other bones with subarachnoid, subdural, and extradural hemorrhage, with prolonged [more than 24 hours] loss of consciousness, without return to pre-existing conscious level
80426	Closed fractures involving skull or face with other bones with subarachnoid, subdural, and extradural hemorrhage, with loss of consciousness of unspecified duration

80429	Closed fractures involving skull or face with other bones with subarachnoid, subdural, and extradural hemorrhage, with concussion, unspecified
80430	Closed fractures involving skull or face with other bones, with other and unspecified intracranial hemorrhage, unspecified state of consciousness
80431	Closed fractures involving skull or face with other bones, with other and unspecified intracranial hemorrhage, with no loss of consciousness
80432	Closed fractures involving skull or face with other bones, with other and unspecified intracranial hemorrhage, with brief [less than one hour] loss of consciousness
80433	Closed fractures involving skull or face with other bones, with other and unspecified intracranial hemorrhage, with moderate [1-24 hours] loss of consciousness
80434	Closed fractures involving skull or face with other bones, with other and unspecified intracranial hemorrhage, with prolonged [more than 24 hours] loss of consciousness and return to pre- existing conscious level
80435	Closed fractures involving skull or face with other bones, with other and unspecified intracranial hemorrhage, with prolonged [more than 24 hours] loss of consciousness, without return to pre-existing conscious level
80436	Closed fractures involving skull or face with other bones, with other and unspecified intracranial hemorrhage, with loss of consciousness of unspecified duration
80439	Closed fractures involving skull or face with other bones, with other and unspecified intracranial hemorrhage, with concussion, unspecified
80440	Closed fractures involving skull or face with other bones, with intracranial injury of other and unspecified nature, unspecified state of consciousness
80441	Closed fractures involving skull or face with other bones, with intracranial injury of other and unspecified nature, with no loss of consciousness
80442	Closed fractures involving skull or face with other bones, with intracranial injury of other and unspecified nature, with brief [less than one hour] loss of consciousness
80443	Closed fractures involving skull or face with other bones, with intracranial injury of other and unspecified nature, with moderate [1-24 hours] loss of consciousness
80444	Closed fractures involving skull or face with other bones, with intracranial injury of other and unspecified nature, with prolonged [more than 24 hours] loss of consciousness and return to pre-existing conscious level
80445	Closed fractures involving skull or face with other bones, with intracranial injury of other and unspecified nature, with prolonged [more than 24 hours] loss of consciousness, without return to pre-existing conscious level
80446	Closed fractures involving skull or face with other bones, with intracranial injury of other and unspecified nature, with loss of consciousness of unspecified duration
80449	Closed fractures involving skull or face with other bones, with intracranial injury of other and unspecified nature, with concussion, unspecified
80450	Open fractures involving skull or face with other bones, without mention of intracranial injury, unspecified state of consciousness
80451	Open fractures involving skull or face with other bones, without mention of intracranial injury, with no loss of consciousness
80452	Open fractures involving skull or face with other bones, without mention of intracranial injury, with brief [less than one hour] loss of consciousness
80453	Open fractures involving skull or face with other bones, without mention of intracranial injury, with moderate [1-24 hours] loss of consciousness

80454	Open fractures involving skull or face with other bones, without mention of intracranial injury, with prolonged [more than 24 hours] loss of consciousness and return to pre-existing conscious level
80455	Open fractures involving skull or face with other bones, without mention of intracranial injury, with prolonged [more than 24 hours] loss of consciousness, without return to pre-existing conscious level
80456	Open fractures involving skull or face with other bones, without mention of intracranial injury, with loss of consciousness of unspecified duration
80459	Open fractures involving skull or face with other bones, without mention of intracranial injury, with concussion, unspecified
80460	Open fractures involving skull or face with other bones, with cerebral laceration and contusion, unspecified state of consciousness
80461	Open fractures involving skull or face with other bones, with cerebral laceration and contusion, with no loss of consciousness
80462	Open fractures involving skull or face with other bones, with cerebral laceration and contusion, with brief [less than one hour] loss of consciousness
80463	Open fractures involving skull or face with other bones, with cerebral laceration and contusion, with moderate [1-24 hours] loss of consciousness
80464	Open fractures involving skull or face with other bones, with cerebral laceration and contusion, with prolonged [more than 24 hours] loss of consciousness and return to pre-existing conscious level
80465	Open fractures involving skull or face with other bones, with cerebral laceration and contusion, with prolonged [more than 24 hours] loss of consciousness, without return to pre-existing conscious level
80466	Open fractures involving skull or face with other bones, with cerebral laceration and contusion, with loss of consciousness of unspecified duration
80469	Open fractures involving skull or face with other bones, with cerebral laceration and contusion, with concussion, unspecified
80470	Open fractures involving skull or face with other bones with subarachnoid, subdural, and extradural hemorrhage, unspecified state of consciousness
80471	Open fractures involving skull or face with other bones with subarachnoid, subdural, and extradural hemorrhage, with no loss of consciousness
80472	Open fractures involving skull or face with other bones with subarachnoid, subdural, and extradural hemorrhage, with brief [less than one hour] loss of consciousness
80473	Open fractures involving skull or face with other bones with subarachnoid, subdural, and extradural hemorrhage, with moderate [1-24 hours] loss of consciousness
80474	Open fractures involving skull or face with other bones with subarachnoid, subdural, and extradural hemorrhage, with prolonged [more than 24 hours] loss of consciousness and return to pre-existing conscious level
80475	Open fractures involving skull or face with other bones with subarachnoid, subdural, and extradural hemorrhage, with prolonged [more than 24 hours] loss of consciousness, without return to pre-existing conscious level
80476	Open fractures involving skull or face with other bones with subarachnoid, subdural, and extradural hemorrhage, with loss of consciousness of unspecified duration
80479	Open fractures involving skull or face with other bones with subarachnoid, subdural, and extradural hemorrhage, with concussion, unspecified

80480	Open fractures involving skull or face with other bones, with other and unspecified intracranial hemorrhage, unspecified state of consciousness
80481	Open fractures involving skull or face with other bones, with other and unspecified intracranial hemorrhage, with no loss of consciousness
80482	Open fractures involving skull or face with other bones, with other and unspecified intracranial hemorrhage, with brief [less than one hour] loss of consciousness
80483	Open fractures involving skull or face with other bones, with other and unspecified intracranial hemorrhage, with moderate [1-24 hours] loss of consciousness
80484	Open fractures involving skull or face with other bones, with other and unspecified intracranial hemorrhage, with prolonged [more than 24 hours] loss of consciousness and return to pre-existing conscious level
80485	Open fractures involving skull or face with other bones, with other and unspecified intracranial hemorrhage, with prolonged [more than 24 hours] loss consciousness, without return to pre-existing conscious level
80486	Open fractures involving skull or face with other bones, with other and unspecified intracranial hemorrhage, with loss of consciousness of unspecified duration
80489	Open fractures involving skull or face with other bones, with other and unspecified intracranial hemorrhage, with concussion, unspecified
80490	Open fractures involving skull or face with other bones, with intracranial injury of other and unspecified nature, unspecified state of consciousness
80491	Open fractures involving skull or face with other bones, with intracranial injury of other and unspecified nature, with no loss of consciousness
80492	Open fractures involving skull or face with other bones, with intracranial injury of other and unspecified nature, with brief [less than one hour] loss of consciousness
80493	Open fractures involving skull or face with other bones, with intracranial injury of other and unspecified nature, with moderate [1-24 hours] loss of consciousness
80494	Open fractures involving skull or face with other bones, with intracranial injury of other and unspecified nature, with prolonged [more than 24 hours] loss of consciousness and return to pre-existing conscious level
80495	Open fractures involving skull or face with other bones, with intracranial injury of other and unspecified nature, with prolonged [more than 24 hours] loss of consciousness without return to pre-existing conscious level
80496	Open fractures involving skull or face with other bones, with intracranial injury of other and unspecified nature, with loss of consciousness of unspecified duration
80499	Open fractures involving skull or face with other bones, with intracranial injury of other and unspecified nature, with concussion, unspecified
8500	Concussion with no loss of consciousness
85011	Concussion, with loss of consciousness of 30 minutes or less
85012	Concussion, with loss of consciousness from 31 to 59 minutes
8502	Concussion with moderate loss of consciousness
8503	Concussion with prolonged loss of consciousness and return to pre-existing conscious level
8504	Concussion with prolonged loss of consciousness, without return to pre-existing conscious level
8505	Concussion with loss of consciousness of unspecified duration
8509	Concussion, unspecified

85100	Cortex (cerebral) contusion without mention of open intracranial wound, unspecified state of consciousness
85101	Cortex (cerebral) contusion without mention of open intracranial wound, with no loss of consciousness
85102	Cortex (cerebral) contusion without mention of open intracranial wound, with brief [less than one hour] loss of consciousness
85103	Cortex (cerebral) contusion without mention of open intracranial wound, with moderate [1-24 hours] loss of consciousness
85104	Cortex (cerebral) contusion without mention of open intracranial wound, with prolonged [more than 24 hours] loss of consciousness and return to pre-existing conscious level
85105	Cortex (cerebral) contusion without mention of open intracranial wound, with prolonged [more than 24 hours] loss of consciousness without return to pre-existing conscious level
85106	Cortex (cerebral) contusion without mention of open intracranial wound, with loss of consciousness of unspecified duration
85109	Cortex (cerebral) contusion without mention of open intracranial wound, with concussion, unspecified
85110	Cortex (cerebral) contusion with open intracranial wound, unspecified state of consciousness
85111	Cortex (cerebral) contusion with open intracranial wound, with no loss of consciousness
85112	Cortex (cerebral) contusion with open intracranial wound, with brief [less than one hour] loss of consciousness
85113	Cortex (cerebral) contusion with open intracranial wound, with moderate [1-24 hours] loss of consciousness
85114	Cortex (cerebral) contusion with open intracranial wound, with prolonged [more than 24 hours] loss of consciousness and return to pre-existing conscious level
85115	Cortex (cerebral) contusion with open intracranial wound, with prolonged [more than 24 hours] loss of consciousness without return to pre-existing conscious level
85116	Cortex (cerebral) contusion with open intracranial wound, with loss of consciousness of unspecified duration
85119	Cortex (cerebral) contusion with open intracranial wound, with concussion, unspecified
85120	Cortex (cerebral) laceration without mention of open intracranial wound, unspecified state of consciousness
85121	Cortex (cerebral) laceration without mention of open intracranial wound, with no loss of consciousness
85122	Cortex (cerebral) laceration without mention of open intracranial wound, with brief [less than one hour] loss of consciousness
85123	Cortex (cerebral) laceration without mention of open intracranial wound, with moderate [1-24 hours] loss of consciousness
85124	Cortex (cerebral) laceration without mention of open intracranial wound, with prolonged [more than 24 hours] loss of consciousness and return to pre-existing conscious level
85125	Cortex (cerebral) laceration without mention of open intracranial wound, with prolonged [more than 24 hours] loss of consciousness without return to pre-existing conscious level
85126	Cortex (cerebral) laceration without mention of open intracranial wound, with loss of consciousness of unspecified duration

85129	Cortex (cerebral) laceration without mention of open intracranial wound, with concussion, unspecified
85130	Cortex (cerebral) laceration with open intracranial wound, unspecified state of consciousness
85131	Cortex (cerebral) laceration with open intracranial wound, with no loss of consciousness
85132	Cortex (cerebral) laceration with open intracranial wound, with brief [less than one hour] loss of consciousness
85133	Cortex (cerebral) laceration with open intracranial wound, with moderate [1-24 hours] loss of consciousness
85134	Cortex (cerebral) laceration with open intracranial wound, with prolonged [more than 24 hours] loss of consciousness and return to pre-existing conscious level
85135	Cortex (cerebral) laceration with open intracranial wound, with prolonged [more than 24 hours] loss of consciousness without return to pre-existing conscious level
85136	Cortex (cerebral) laceration with open intracranial wound, with loss of consciousness of unspecified duration
85139	Cortex (cerebral) laceration with open intracranial wound, with concussion, unspecified
85140	Cerebellar or brain stem contusion without mention of open intracranial wound, unspecified state of consciousness
85141	Cerebellar or brain stem contusion without mention of open intracranial wound, with no loss of consciousness
85142	Cerebellar or brain stem contusion without mention of open intracranial wound, with brief [less than one hour] loss of consciousness
85143	Cerebellar or brain stem contusion without mention of open intracranial wound, with moderate [1-24 hours] loss of consciousness
85144	Cerebellar or brain stem contusion without mention of open intracranial wound, with prolonged [more than 24 hours] loss consciousness and return to pre-existing conscious level
85145	Cerebellar or brain stem contusion without mention of open intracranial wound, with prolonged [more than 24 hours] loss of consciousness without return to pre-existing conscious level
85146	Cerebellar or brain stem contusion without mention of open intracranial wound, with loss of consciousness of unspecified duration
85149	Cerebellar or brain stem contusion without mention of open intracranial wound, with concussion, unspecified
85150	Cerebellar or brain stem contusion with open intracranial wound, unspecified state of consciousness
85151	Cerebellar or brain stem contusion with open intracranial wound, with no loss of consciousness
85152	Cerebellar or brain stem contusion with open intracranial wound, with brief [less than one hour] loss of consciousness
85153	Cerebellar or brain stem contusion with open intracranial wound, with moderate [1-24 hours] loss of consciousness
85154	Cerebellar or brain stem contusion with open intracranial wound, with prolonged [more than 24 hours] loss of consciousness and return to pre-existing conscious level

85155	Cerebellar or brain stem contusion with open intracranial wound, with prolonged [more than 24 hours] loss of consciousness without return to pre-existing conscious level
85156	Cerebellar or brain stem contusion with open intracranial wound, with loss of consciousness of unspecified duration
85159	Cerebellar or brain stem contusion with open intracranial wound, with concussion, unspecified
85160	Cerebellar or brain stem laceration without mention of open intracranial wound, unspecified state of consciousness
85161	Cerebellar or brain stem laceration without mention of open intracranial wound, with no loss of consciousness
85162	Cerebellar or brain stem laceration without mention of open intracranial wound, with brief [less than 1 hour] loss of consciousness
85163	Cerebellar or brain stem laceration without mention of open intracranial wound, with moderate [1-24 hours] loss of consciousness
85164	Cerebellar or brain stem laceration without mention of open intracranial wound, with prolonged [more than 24 hours] loss of consciousness and return to pre-existing conscious level
85165	Cerebellar or brain stem laceration without mention of open intracranial wound, with prolonged [more than 24 hours] loss of consciousness without return to pre-existing conscious level
85166	Cerebellar or brain stem laceration without mention of open intracranial wound, with loss of consciousness of unspecified duration
85169	Cerebellar or brain stem laceration without mention of open intracranial wound, with concussion, unspecified
85170	Cerebellar or brain stem laceration with open intracranial wound, unspecified state of consciousness
85171	Cerebellar or brain stem laceration with open intracranial wound, with no loss of consciousness
85172	Cerebellar or brain stem laceration with open intracranial wound, with brief [less than one hour] loss of consciousness
85173	Cerebellar or brain stem laceration with open intracranial wound, with moderate [1-24 hours] loss of consciousness
85174	Cerebellar or brain stem laceration with open intracranial wound, with prolonged [more than 24 hours] loss of consciousness and return to pre-existing conscious level
85175	Cerebellar or brain stem laceration with open intracranial wound, with prolonged [more than 24 hours] loss of consciousness without return to pre-existing conscious level
85176	Cerebellar or brain stem laceration with open intracranial wound, with loss of consciousness of unspecified duration
85179	Cerebellar or brain stem laceration with open intracranial wound, with concussion, unspecified
85180	Other and unspecified cerebral laceration and contusion, without mention of open intracranial wound, unspecified state of consciousness
85181	Other and unspecified cerebral laceration and contusion, without mention of open intracranial wound, with no loss of consciousness
85182	Other and unspecified cerebral laceration and contusion, without mention of open intracranial wound, with brief [less than one hour] loss of consciousness

85183	Other and unspecified cerebral laceration and contusion, without mention of open intracranial wound, with moderate [1-24 hours] loss of consciousness
85184	Other and unspecified cerebral laceration and contusion, without mention of open intracranial wound, with prolonged [more than 24 hours] loss of consciousness and return to pre- existing conscious level
85185	Other and unspecified cerebral laceration and contusion, without mention of open intracranial wound, with prolonged [more than 24 hours] loss of consciousness without return to pre-existing conscious level
85186	Other and unspecified cerebral laceration and contusion, without mention of open intracranial wound, with loss of consciousness of unspecified duration
85189	Other and unspecified cerebral laceration and contusion, without mention of open intracranial wound, with concussion, unspecified
85190	Other and unspecified cerebral laceration and contusion, with open intracranial wound, unspecified state of consciousness
85191	Other and unspecified cerebral laceration and contusion, with open intracranial wound, with no loss of consciousness
85192	Other and unspecified cerebral laceration and contusion, with open intracranial wound, with brief [less than one hour] loss of consciousness
85193	Other and unspecified cerebral laceration and contusion, with open intracranial wound, with moderate [1-24 hours] loss of consciousness
85194	Other and unspecified cerebral laceration and contusion, with open intracranial wound, with prolonged [more than 24 hours] loss of consciousness and return to pre-existing conscious level
85195	Other and unspecified cerebral laceration and contusion, with open intracranial wound, with prolonged [more than 24 hours] loss of consciousness without return to pre-existing conscious level
85196	Other and unspecified cerebral laceration and contusion, with open intracranial wound, with loss of consciousness of unspecified duration
85199	Other and unspecified cerebral laceration and contusion, with open intracranial wound, with concussion, unspecified
85200	Subarachnoid hemorrhage following injury without mention of open intracranial wound, unspecified state of consciousness
85201	Subarachnoid hemorrhage following injury without mention of open intracranial wound, with no loss of consciousness
85202	Subarachnoid hemorrhage following injury without mention of open intracranial wound, with brief [less than one hour] loss of consciousness
85203	Subarachnoid hemorrhage following injury without mention of open intracranial wound, with moderate [1-24 hours] loss of consciousness
85204	Subarachnoid hemorrhage following injury without mention of open intracranial wound, with prolonged [more than 24 hours] loss of consciousness and return to pre-existing conscious level
85205	Subarachnoid hemorrhage following injury without mention of open intracranial wound, with prolonged [more than 24 hours] loss of consciousness without return to pre-existing conscious level
85206	Subarachnoid hemorrhage following injury without mention of open intracranial wound, with loss of consciousness of unspecified duration

85209	Subarachnoid hemorrhage following injury without mention of open intracranial wound, with concussion, unspecified
85210	Subarachnoid hemorrhage following injury with open intracranial wound, unspecified state of consciousness
85211	Subarachnoid hemorrhage following injury with open intracranial wound, with no loss of consciousness
85212	Subarachnoid hemorrhage following injury with open intracranial wound, with brief [less than one hour] loss of consciousness
85213	Subarachnoid hemorrhage following injury with open intracranial wound, with moderate [1-24 hours] loss of consciousness
85214	Subarachnoid hemorrhage following injury with open intracranial wound, with prolonged [more than 24 hours) loss of consciousness and return to pre-existing conscious level
85215	Subarachnoid hemorrhage following injury with open intracranial wound, with prolonged [more than 24 hours] loss of consciousness without return to pre-existing conscious level
85216	Subarachnoid hemorrhage following injury with open intracranial wound, with loss of consciousness of unspecified duration
85219	Subarachnoid hemorrhage following injury with open intracranial wound, with concussion, unspecified
85220	Subdural hemorrhage following injury without mention of open intracranial wound, unspecified state of consciousness
85221	Subdural hemorrhage following injury without mention of open intracranial wound, with no loss of consciousness
85222	Subdural hemorrhage following injury without mention of open intracranial wound, with brief [less than one hour] loss of consciousness
85223	Subdural hemorrhage following injury without mention of open intracranial wound, with moderate [1-24 hours] loss of consciousness
85224	Subdural hemorrhage following injury without mention of open intracranial wound, with prolonged [more than 24 hours] loss of consciousness and return to pre-existing conscious level
85225	Subdural hemorrhage following injury without mention of open intracranial wound, with prolonged [more than 24 hours] loss of consciousness without return to pre-existing conscious level
85226	Subdural hemorrhage following injury without mention of open intracranial wound, with loss of consciousness of unspecified duration
85229	Subdural hemorrhage following injury without mention of open intracranial wound, with concussion, unspecified
85230	Subdural hemorrhage following injury with open intracranial wound, unspecified state of consciousness
85231	Subdural hemorrhage following injury with open intracranial wound, with no loss of consciousness
85232	Subdural hemorrhage following injury with open intracranial wound, with brief [less than one hour] loss of consciousness
85233	Subdural hemorrhage following injury with open intracranial wound, with moderate [1-24 hours] loss of consciousness
85234	Subdural hemorrhage following injury with open intracranial wound, with prolonged [more than 24 hours] loss of consciousness and return to pre-existing conscious level

85235	Subdural hemorrhage following injury with open intracranial wound, with prolonged [more than 24 hours] loss of consciousness without return to pre-existing conscious level
85236	Subdural hemorrhage following injury with open intracranial wound, with loss of consciousness of unspecified duration
85239	Subdural hemorrhage following injury with open intracranial wound, with concussion, unspecified
85240	Extradural hemorrhage following injury without mention of open intracranial wound, unspecified state of consciousness
85241	Extradural hemorrhage following injury without mention of open intracranial wound, with no loss of consciousness
85242	Extradural hemorrhage following injury without mention of open intracranial wound, with brief [less than 1 hour] loss of consciousness
85243	Extradural hemorrhage following injury without mention of open intracranial wound, with moderate [1-24 hours] loss of consciousness
85244	Extradural hemorrhage following injury without mention of open intracranial wound, with prolonged [more than 24 hours] loss of consciousness and return to pre-existing conscious level
85245	Extradural hemorrhage following injury without mention of open intracranial wound, with prolonged [more than 24 hours] loss of consciousness without return to pre-existing conscious level
85246	Extradural hemorrhage following injury without mention of open intracranial wound, with loss of consciousness of unspecified duration
85249	Extradural hemorrhage following injury without mention of open intracranial wound, with concussion, unspecified
85250	Extradural hemorrhage following injury with open intracranial wound, unspecified state of consciousness
85251	Extradural hemorrhage following injury with open intracranial wound, with no loss of consciousness
85252	Extradural hemorrhage following injury with open intracranial wound, with brief [less than one hour] loss of consciousness
85253	Extradural hemorrhage following injury with open intracranial wound, with moderate [1-24 hours] loss of consciousness
85254	Extradural hemorrhage following injury with open intracranial wound, with prolonged [more than 24 hours] loss of consciousness and return to pre-existing conscious level
85255	Extradural hemorrhage following injury with open intracranial wound, with prolonged [more than 24 hours] loss of consciousness without return to pre-existing conscious level
85256	Extradural hemorrhage following injury with open intracranial wound, with loss of consciousness of unspecified duration
85259	Extradural hemorrhage following injury with open intracranial wound, with concussion, unspecified
85300	Other and unspecified intracranial hemorrhage following injury without mention of open intracranial wound, unspecified state of consciousness
85301	Other and unspecified intracranial hemorrhage following injury without mention of open intracranial wound, with no loss of consciousness
85302	Other and unspecified intracranial hemorrhage following injury without mention of open intracranial wound, with brief [less than one hour] loss of consciousness

85303	Other and unspecified intracranial hemorrhage following injury without mention of open intracranial wound, with moderate [1-24 hours] loss of consciousness
85304	Other and unspecified intracranial hemorrhage following injury without mention of open intracranial wound, with prolonged [more than 24 hours] loss of consciousness and return to pre- existing conscious level
85305	Other and unspecified intracranial hemorrhage following injury without mention of open intracranial wound, with prolonged [more than 24 hours] loss of consciousness without return to pre-existing conscious level
85306	Other and unspecified intracranial hemorrhage following injury without mention of open intracranial wound, with loss of consciousness of unspecified duration
85309	Other and unspecified intracranial hemorrhage following injury without mention of open intracranial wound, with concussion, unspecified
85310	Other and unspecified intracranial hemorrhage following injury with open intracranial wound, unspecified state of consciousness
85311	Other and unspecified intracranial hemorrhage following injury with open intracranial wound, with no loss of consciousness
85312	Other and unspecified intracranial hemorrhage following injury with open intracranial wound, with brief [less than one hour] loss of consciousness
85313	Other and unspecified intracranial hemorrhage following injury with open intracranial wound, with moderate [1-24 hours] loss of consciousness
85314	Other and unspecified intracranial hemorrhage following injury with open intracranial wound, with prolonged [more than 24 hours] loss of consciousness and return to pre-existing conscious level
85315	Other and unspecified intracranial hemorrhage following injury with open intracranial wound, with prolonged [more than 24 hours] loss of consciousness without return to pre-existing conscious level
85316	Other and unspecified intracranial hemorrhage following injury with open intracranial wound, with loss of consciousness of unspecified duration
85319	Other and unspecified intracranial hemorrhage following injury with open intracranial wound, with concussion, unspecified
85400	Intracranial injury of other and unspecified nature without mention of open intracranial wound, unspecified state of consciousness
85401	Intracranial injury of other and unspecified nature without mention of open intracranial wound, with no loss of consciousness
85402	Intracranial injury of other and unspecified nature without mention of open intracranial wound, with brief [less than one hour] loss of consciousness
85403	Intracranial injury of other and unspecified nature without mention of open intracranial wound, with moderate [1-24 hours] loss of consciousness
85404	Intracranial injury of other and unspecified nature without mention of open intracranial wound, with prolonged [more than 24 hours] loss of consciousness and return to pre-existing conscious level
85405	Intracranial injury of other and unspecified nature without mention of open intracranial wound, with prolonged [more than 24 hours] loss of consciousness without return to pre-existing conscious level
85406	Intracranial injury of other and unspecified nature without mention of open intracranial wound, with loss of consciousness of unspecified duration

85409	Intracranial injury of other and unspecified nature without mention of open intracranial wound, with concussion, unspecified
85410	Intracranial injury of other and unspecified nature with open intracranial wound, unspecified state of consciousness
85411	Intracranial injury of other and unspecified nature with open intracranial wound, with no loss of consciousness
85412	Intracranial injury of other and unspecified nature with open intracranial wound, with brief [less than one hour] loss of consciousness
85413	Intracranial injury of other and unspecified nature with open intracranial wound, with moderate [1-24 hours] loss of consciousness
85414	Intracranial injury of other and unspecified nature with open intracranial wound, with prolonged [more than 24 hours] loss of consciousness and return to pre-existing conscious level
85415	Intracranial injury of other and unspecified nature with open intracranial wound, with prolonged [more than 24 hours] loss of consciousness without return to pre-existing conscious level
85416	Intracranial injury of other and unspecified nature with open intracranial wound, with loss of consciousness of unspecified duration
85419	Intracranial injury of other and unspecified nature with open intracranial wound, with concussion, unspecified
99555	Shaken baby syndrome

ICD 10 codes SWD

F5101	Primary insomnia
F5101	Adjustment insomnia
F5103	Paradoxical insomnia
F5104	Psychophysiological insomnia
F5105	Insomnia due to other mental disorder
F5109	Other insomnia not due to a substance or known physiological condition
F5111	Primary hypersomnia
F5112	Insufficient sleep syndrome
F5113	Hypersomnia due to other mental disorder
F5119	Other hypersomnia not due to a substance or known physiological condition
F513	Sleepwalking (somnambulism)
F514	Sleep terrors (night terrors)
F515	Nightmare disorder
F518	Other sleep disorders not due to a substances or known physiological condition
F519	Sleep disorder not due to a substance or known physiological condition, unspecified
G4700	Insomnia, unspecified
G4709	Other insomnia
G4711	Idiopathic hypersomnia with long sleep time
G4712	Idiopathic hypersomnia without a long sleep time

G4713	Recurrent hypersomnia
G4714	Other hypersomnia due to medical condition
G4719	Other hypersomnia
G4720	Circadian rhythm sleep disorder, unspecified type
G4721	Circadian rhythm sleep disorder, delayed sleep phase type
G4722	Circadian rhythm sleep disorder, advanced sleep phase type
G4723	Circadian rhythm sleep disorder, irregular sleep wake type
G4724	Circadian rhythm sleep disorder, free running type
G4725	Circadian rhythm sleep disorder, jet lag type
G4726	Circadian rhythm sleep disorder, shift work type
G4727	Circadian rhythm sleep disorder in conditions classified elsewhere
G4729	Other circadian rhythm sleep disorder
G4730	Sleep apnea, unspecified
G4731	Primary central sleep apnea
G4732	High altitude periodic breathing
G4733	Obstructive sleep apnea (adult) (pediatric)
G4734	Idiopathic sleep related nonobstructive alveolar hypoventilation
G4735	Congenital central alveolar hypoventilation syndrome
G4736	Sleep related hypoventilation in conditions classified elsewhere
G4737	Central sleep apnea in conditions classified elsewhere
G4739	Other sleep apnea
G47411	Narcolepsy with cataplexy
G47419	Narcolepsy without cataplexy
G47421	Narcolepsy in conditions classified elsewhere with cataplexy
G47429	Narcolepsy in conditions classified elsewhere without cataplexy
G4750	Parasomnia, unspecified
G4751	Confusional arousals
G4752	REM sleep behavior disorder
G4753	Recurrent isolated sleep paralysis
G4754	Parasomnia in conditions classified elsewhere
G4759	Other parasomnia
G4761	Periodic limb movement disorder
G4762	Sleep related leg cramps
G4763	Sleep related bruxism
G4769	Other sleep related movement disorders
G478	Other sleep disorders
G479	disorder, unspecified

ICD 10 Codes TBI

R4020	Unspecified coma
R402110	Coma scale, eyes open, never, unspecified time

R402111	Coma scale, eyes open, never, in the field [EMT or ambulance]
R402112	Coma scale, eyes open, never, at arrival to emergency department
R402113	Coma scale, eyes open, never, at hospital admission
R402114	Coma scale, eyes open, never, 24 hours or more after hospital admission
R402120	Coma scale, eyes open, to pain, unspecified time
R402121	Coma scale, eyes open, to pain, in the field [EMT or ambulance]
R402122	Coma scale, eyes open, to pain, at arrival to emergency department
R402123	Coma scale, eyes open, to pain, at hospital admission
R402124	Coma scale, eyes open, to pain, 24 hours or more after hospital admission
R402130	Coma scale, eyes open, to sound, unspecified time
R402131	Coma scale, eyes open, to sound, in the field [EMT or ambulance]
R402132	Coma scale, eyes open, to sound, at arrival to emergency department
R402133	Coma scale, eyes open, to sound, at hospital admission
R402134	Coma scale, eyes open, to sound, 24 hours or more after hospital admission
R402140	Coma scale, eyes open, spontaneous, unspecified time
R402141	Coma scale, eyes open, spontaneous, in the field [EMT or ambulance]
R402142	Coma scale, eyes open, spontaneous, at arrival to emergency department
R402143	Coma scale, eyes open, spontaneous, at hospital admission
R402144	Coma scale, eyes open, spontaneous, 24 hours or more after hospital admission
R402210	Coma scale, best verbal response, none, unspecified time
R402211	Coma scale, best verbal response, none, in the field [EMT or ambulance]
R402212	Coma scale, best verbal response, none, at arrival to emergency department
R402213	Coma scale, best verbal response, none, at hospital admission
R402214	Coma scale, best verbal response, none, 24 hours or more after hospital admission
R402220	Coma scale, best verbal response, incomprehensible words, unspecified time
R402221	Coma scale, best verbal response, incomprehensible words, in the field [EMT or ambulance]
R402222	Coma scale, best verbal response, incomprehensible words, at arrival to emergency department
R402223	Coma scale, best verbal response, incomprehensible words, at hospital admission
R402224	Coma scale, best verbal response, incomprehensible words, 24 hours or more after hospital admission
R402230	Coma scale, best verbal response, inappropriate words, unspecified time
R402231	Coma scale, best verbal response, inappropriate words, in the field [EMT or ambulance]
R402232	Coma scale, best verbal response, inappropriate words, at arrival to emergency department
R402233	Coma scale, best verbal response, inappropriate words, at hospital admission
R402234	Coma scale, best verbal response, inappropriate words, 24 hours or more after hospital admission
R402240	Coma scale, best verbal response, confused conversation, unspecified time
R402241	Coma scale, best verbal response, confused conversation, in the field [EMT or ambulance]
R402242	Coma scale, best verbal response, confused conversation, at arrival to emergency department

R402243	Coma scale, best verbal response, confused conversation, at hospital admission
R402244	Coma scale, best verbal response, confused conversation, 24 hours or more after hospital admission
R402250	Coma scale, best verbal response, oriented, unspecified time
R402251	Coma scale, best verbal response, oriented, in the field [EMT or ambulance]
R402252	Coma scale, best verbal response, oriented, at arrival to emergency department
R402253	Coma scale, best verbal response, oriented, at hospital admission
R402254	Coma scale, best verbal response, oriented, 24 hours or more after hospital admission
R402310	Coma scale, best motor response, none, unspecified time
R402311	Coma scale, best motor response, none, in the field [EMT or ambulance]
R402312	Coma scale, best motor response, none, at arrival to emergency department
R402313	Coma scale, best motor response, none, at hospital admission
R402314	Coma scale, best motor response, none, 24 hours or more after hospital admission
R402320	Coma scale, best motor response, extension, unspecified time
R402321	Coma scale, best motor response, extension, in the field [EMT or ambulance]
R402322	Coma scale, best motor response, extension, at arrival to emergency department
R402323	Coma scale, best motor response, extension, at hospital admission
R402324	Coma scale, best motor response, extension, 24 hours or more after hospital admission
R402330	Coma scale, best motor response, abnormal flexion, unspecified time
R402331	Coma scale, best motor response, abnormal flexion, in the field [EMT or ambulance]
R402332	Coma scale, best motor response, abnormal flexion, at arrival to emergency department
R402333	Coma scale, best motor response, abnormal flexion, at hospital admission
R402334	Coma scale, best motor response, abnormal flexion, 24 hours or more after hospital admission
R402340	Coma scale, best motor response, flexion withdrawal, unspecified time
R402341	Coma scale, best motor response, flexion withdrawal, in the field [EMT or ambulance]
R402342	Coma scale, best motor response, flexion withdrawal, at arrival to emergency department
R402343	Coma scale, best motor response, flexion withdrawal, at hospital admission
R402344	Coma scale, best motor response, flexion withdrawal, 24 hours or more after hospital admission
R402350	Coma scale, best motor response, localizes pain, unspecified time
R402351	Coma scale, best motor response, localizes pain, in the field [EMT or ambulance]
R402352	Coma scale, best motor response, localizes pain, at arrival to emergency department
R402353	Coma scale, best motor response, localizes pain, at hospital admission
R402354	Coma scale, best motor response, localizes pain, 24 hours or more after hospital admission
R402360	Coma scale, best motor response, obeys commands, unspecified time
R402361	Coma scale, best motor response, obeys commands, in the field [EMT or ambulance]
R402362	Coma scale, best motor response, obeys commands, at arrival to emergency department
R402363	Coma scale, best motor response, obeys commands, at hospital admission
R402364	Coma scale, best motor response, obeys commands, 24 hours or more after hospital admission
R402410	Glasgow coma scale score 13-15, unspecified time

R402411	Glasgow coma scale score 13-15, in the field [EMT or ambulance]
R402412	Glasgow coma scale score 13-15, at arrival to emergency department
R402413	Glasgow coma scale score 13-15, at hospital admission
R402414	Glasgow coma scale score 13-15, 24 hours or more after hospital admission
R402420	Glasgow coma scale score 9-12, unspecified time
R402421	Glasgow coma scale score 9-12, in the field [EMT or ambulance]
R402422	Glasgow coma scale score 9-12, at arrival to emergency department
R402423	Glasgow coma scale score 9-12, at hospital admission
R402424	Glasgow coma scale score 9-12, 24 hours or more after hospital admission
R402430	Glasgow coma scale score 3-8, unspecified time
R402431	Glasgow coma scale score 3-8, in the field [EMT or ambulance]
R402432	Glasgow coma scale score 3-8, at arrival to emergency department
R402433	Glasgow coma scale score 3-8, at hospital admission
R402434	Glasgow coma scale score 3-8, 24 hours or more after hospital admission
R402440	Other coma, without documented Glasgow coma scale score, or with partial score reported, unspecified time
R402441	Other coma, without documented Glasgow coma scale score, or with partial score reported, in the field [EMT or ambulance]
R402442	Other coma, without documented Glasgow coma scale score, or with partial score reported, at arrival to emergency department
R402443	Other coma, without documented Glasgow coma scale score, or with partial score reported, at hospital admission
R402444	Other coma, without documented Glasgow coma scale score, or with partial score reported, 24 hours or more after hospital admission
S060X0A	Concussion without loss of consciousness, initial encounter
S060X0D	Concussion without loss of consciousness, subsequent encounter
S060X0S	Concussion without loss of consciousness, sequela
S060X1A	Concussion with loss of consciousness of 30 minutes or less, initial encounter
S060X1D	Concussion with loss of consciousness of 30 minutes or less, subsequent encounter
S060X1S	Concussion with loss of consciousness of 30 minutes or less, sequela
S060X9A	Concussion with loss of consciousness of unspecified duration, initial encounter
S060X9D	Concussion with loss of consciousness of unspecified duration, subsequent encounter
S060X9S	Concussion with loss of consciousness of unspecified duration, sequela
S061X0A	Traumatic cerebral edema without loss of consciousness, initial encounter
S061X0D	Traumatic cerebral edema without loss of consciousness, subsequent encounter
S061X0S	Traumatic cerebral edema without loss of consciousness, sequela
S061X1A	Traumatic cerebral edema with loss of consciousness of 30 minutes or less, initial encounter
S061X1D	Traumatic cerebral edema with loss of consciousness of 30 minutes or less, subsequent encounter
S061X1S	Traumatic cerebral edema with loss of consciousness of 30 minutes or less, sequela

S061X2A	Traumatic cerebral edema with loss of consciousness of 31 minutes to 59 minutes, initial encounter
S061X2D	Traumatic cerebral edema with loss of consciousness of 31 minutes to 59 minutes, subsequent encounter
S061X2S	Traumatic cerebral edema with loss of consciousness of 31 minutes to 59 minutes, sequela
S061X3A	Traumatic cerebral edema with loss of consciousness of 1 hour to 5 hours 59 minutes, initial encounter
S061X3D	Traumatic cerebral edema with loss of consciousness of 1 hour to 5 hours 59 minutes, subsequent encounter
S061X3S	Traumatic cerebral edema with loss of consciousness of 1 hour to 5 hours 59 minutes, sequela
S061X4A	Traumatic cerebral edema with loss of consciousness of 6 hours to 24 hours, initial encounter
S061X4D	Traumatic cerebral edema with loss of consciousness of 6 hours to 24 hours, subsequent encounter
S061X4S	Traumatic cerebral edema with loss of consciousness of 6 hours to 24 hours, sequela
S061X5A	Traumatic cerebral edema with loss of consciousness greater than 24 hours with return to pre-existing conscious level, initial encounter
S061X5D	Traumatic cerebral edema with loss of consciousness greater than 24 hours with return to pre-existing conscious level, subsequent encounter
S061X5S	Traumatic cerebral edema with loss of consciousness greater than 24 hours with return to pre-existing conscious level, sequela
S061X6A	Traumatic cerebral edema with loss of consciousness greater than 24 hours without return to pre-existing conscious level with patient surviving, initial encounter
S061X6D	Traumatic cerebral edema with loss of consciousness greater than 24 hours without return to pre-existing conscious level with patient surviving, subsequent encounter
S061X6S	Traumatic cerebral edema with loss of consciousness greater than 24 hours without return to pre-existing conscious level with patient surviving, sequela
S061X7A	Traumatic cerebral edema with loss of consciousness of any duration with death due to brain injury prior to regaining consciousness, initial encounter
S061X8A	Traumatic cerebral edema with loss of consciousness of any duration with death due to other cause prior to regaining consciousness, initial encounter
S061X9A	Traumatic cerebral edema with loss of consciousness of unspecified duration, initial encounter
S061X9D	Traumatic cerebral edema with loss of consciousness of unspecified duration, subsequent encounter
S061X9S	Traumatic cerebral edema with loss of consciousness of unspecified duration, sequela
S062X0A	Diffuse traumatic brain injury without loss of consciousness, initial encounter
S062X0D	Diffuse traumatic brain injury without loss of consciousness, subsequent encounter
S062X0S	Diffuse traumatic brain injury without loss of consciousness, sequela
S062X1A	Diffuse traumatic brain injury with loss of consciousness of 30 minutes or less, initial encounter
S062X1D	Diffuse traumatic brain injury with loss of consciousness of 30 minutes or less, subsequent encounter
S062X1S	Diffuse traumatic brain injury with loss of consciousness of 30 minutes or less, sequela

S062X2A	Diffuse traumatic brain injury with loss of consciousness of 31 minutes to 59 minutes, initial encounter
S062X2D	Diffuse traumatic brain injury with loss of consciousness of 31 minutes to 59 minutes, subsequent encounter
S062X2S	Diffuse traumatic brain injury with loss of consciousness of 31 minutes to 59 minutes, sequela
S062X3A	Diffuse traumatic brain injury with loss of consciousness of 1 hour to 5 hours 59 minutes, initial encounter
S062X3D	Diffuse traumatic brain injury with loss of consciousness of 1 hour to 5 hours 59 minutes, subsequent encounter
S062X3S	Diffuse traumatic brain injury with loss of consciousness of 1 hour to 5 hours 59 minutes, sequela
S062X4A	Diffuse traumatic brain injury with loss of consciousness of 6 hours to 24 hours, initial encounter
S062X4D	Diffuse traumatic brain injury with loss of consciousness of 6 hours to 24 hours, subsequent encounter
S062X4S	Diffuse traumatic brain injury with loss of consciousness of 6 hours to 24 hours, sequela
S062X5A	Diffuse traumatic brain injury with loss of consciousness greater than 24 hours with return to pre-existing conscious levels, initial encounter
S062X5D	Diffuse traumatic brain injury with loss of consciousness greater than 24 hours with return to pre-existing conscious levels, subsequent encounter
S062X5S	Diffuse traumatic brain injury with loss of consciousness greater than 24 hours with return to pre-existing conscious levels, sequela
S062X6A	Diffuse traumatic brain injury with loss of consciousness greater than 24 hours without return to pre-existing conscious level with patient surviving, initial encounter
S062X6D	Diffuse traumatic brain injury with loss of consciousness greater than 24 hours without return to pre-existing conscious level with patient surviving, subsequent encounter
S062X6S	Diffuse traumatic brain injury with loss of consciousness greater than 24 hours without return to pre-existing conscious level with patient surviving, sequela
S062X7A	Diffuse traumatic brain injury with loss of consciousness of any duration with death due to brain injury prior to regaining consciousness, initial encounter
S062X8A	Diffuse traumatic brain injury with loss of consciousness of any duration with death due to other cause prior to regaining consciousness, initial encounter
S062X9A	Diffuse traumatic brain injury with loss of consciousness of unspecified duration, initial encounter
S062X9D	Diffuse traumatic brain injury with loss of consciousness of unspecified duration, subsequent encounter
S062X9S	Diffuse traumatic brain injury with loss of consciousness of unspecified duration, sequela
S06300A	Unspecified focal traumatic brain injury without loss of consciousness, initial encounter
S06300D	Unspecified focal traumatic brain injury without loss of consciousness, subsequent encounter
S06300S	Unspecified focal traumatic brain injury without loss of consciousness, sequela
S06301A	Unspecified focal traumatic brain injury with loss of consciousness of 30 minutes or less, initial encounter

S06301D	Unspecified focal traumatic brain injury with loss of consciousness of 30 minutes or less, subsequent encounter
S06301S	Unspecified focal traumatic brain injury with loss of consciousness of 30 minutes or less, sequela
S06302A	Unspecified focal traumatic brain injury with loss of consciousness of 31 minutes to 59 minutes, initial encounter
S06302D	Unspecified focal traumatic brain injury with loss of consciousness of 31 minutes to 59 minutes, subsequent encounter
S06302S	Unspecified focal traumatic brain injury with loss of consciousness of 31 minutes to 59 minutes, sequela
S06303A	Unspecified focal traumatic brain injury with loss of consciousness of 1 hour to 5 hours 59 minutes, initial encounter
S06303D	Unspecified focal traumatic brain injury with loss of consciousness of 1 hour to 5 hours 59 minutes, subsequent encounter
S06303S	Unspecified focal traumatic brain injury with loss of consciousness of 1 hour to 5 hours 59 minutes, sequela
S06304A	Unspecified focal traumatic brain injury with loss of consciousness of 6 hours to 24 hours, initial encounter
S06304D	Unspecified focal traumatic brain injury with loss of consciousness of 6 hours to 24 hours, subsequent encounter
S06304S	Unspecified focal traumatic brain injury with loss of consciousness of 6 hours to 24 hours, sequela
S06305A	Unspecified focal traumatic brain injury with loss of consciousness greater than 24 hours with return to pre-existing conscious level, initial encounter
S06305D	Unspecified focal traumatic brain injury with loss of consciousness greater than 24 hours with return to pre-existing conscious level, subsequent encounter
S06305S	Unspecified focal traumatic brain injury with loss of consciousness greater than 24 hours with return to pre-existing conscious level, sequela
S06306A	Unspecified focal traumatic brain injury with loss of consciousness greater than 24 hours without return to pre-existing conscious level with patient surviving, initial encounter
S06306D	Unspecified focal traumatic brain injury with loss of consciousness greater than 24 hours without return to pre-existing conscious level with patient surviving, subsequent encounter
S06306S	Unspecified focal traumatic brain injury with loss of consciousness greater than 24 hours without return to pre-existing conscious level with patient surviving, sequela
S06307A	Unspecified focal traumatic brain injury with loss of consciousness of any duration with death due to brain injury prior to regaining consciousness, initial encounter
S06308A	Unspecified focal traumatic brain injury with loss of consciousness of any duration with death due to other cause prior to regaining consciousness, initial encounter
S06309A	Unspecified focal traumatic brain injury with loss of consciousness of unspecified duration, initial encounter
S06309D	Unspecified focal traumatic brain injury with loss of consciousness of unspecified duration, subsequent encounter
S06309S	Unspecified focal traumatic brain injury with loss of consciousness of unspecified duration, sequela
S06310A	Contusion and laceration of right cerebrum without loss of consciousness, initial encounter

S06310D	Contusion and laceration of right cerebrum without loss of consciousness, subsequent encounter
S06310S	Contusion and laceration of right cerebrum without loss of consciousness, sequela
S06311A	Contusion and laceration of right cerebrum with loss of consciousness of 30 minutes or less, initial encounter
S06311D	Contusion and laceration of right cerebrum with loss of consciousness of 30 minutes or less, subsequent encounter
S06311S	Contusion and laceration of right cerebrum with loss of consciousness of 30 minutes or less, sequela
S06312A	Contusion and laceration of right cerebrum with loss of consciousness of 31 minutes to 59 minutes, initial encounter
S06312D	Contusion and laceration of right cerebrum with loss of consciousness of 31 minutes to 59 minutes, subsequent encounter
S06312S	Contusion and laceration of right cerebrum with loss of consciousness of 31 minutes to 59 minutes, sequela
S06313A	Contusion and laceration of right cerebrum with loss of consciousness of 1 hour to 5 hours 59 minutes, initial encounter
S06313D	Contusion and laceration of right cerebrum with loss of consciousness of 1 hour to 5 hours 59 minutes, subsequent encounter
S06313S	Contusion and laceration of right cerebrum with loss of consciousness of 1 hour to 5 hours 59 minutes, sequela
S06314A	Contusion and laceration of right cerebrum with loss of consciousness of 6 hours to 24 hours, initial encounter
S06314D	Contusion and laceration of right cerebrum with loss of consciousness of 6 hours to 24 hours, subsequent encounter
S06314S	Contusion and laceration of right cerebrum with loss of consciousness of 6 hours to 24 hours, sequela
S06315A	Contusion and laceration of right cerebrum with loss of consciousness greater than 24 hours with return to pre-existing conscious level, initial encounter
S06315D	Contusion and laceration of right cerebrum with loss of consciousness greater than 24 hours with return to pre-existing conscious level, subsequent encounter
S06315S	Contusion and laceration of right cerebrum with loss of consciousness greater than 24 hours with return to pre-existing conscious level, sequela
S06316A	Contusion and laceration of right cerebrum with loss of consciousness greater than 24 hours without return to pre-existing conscious level with patient surviving, initial encounter
S06316D	Contusion and laceration of right cerebrum with loss of consciousness greater than 24 hours without return to pre-existing conscious level with patient surviving, subsequent encounter
S06316S	Contusion and laceration of right cerebrum with loss of consciousness greater than 24 hours without return to pre-existing conscious level with patient surviving, sequela
S06317A	Contusion and laceration of right cerebrum with loss of consciousness of any duration with death due to brain injury prior to regaining consciousness, initial encounter
S06318A	Contusion and laceration of right cerebrum with loss of consciousness of any duration with death due to other cause prior to regaining consciousness, initial encounter

S06319A	Contusion and laceration of right cerebrum with loss of consciousness of unspecified duration, initial encounter
S06319D	Contusion and laceration of right cerebrum with loss of consciousness of unspecified duration, subsequent encounter
S06319S	Contusion and laceration of right cerebrum with loss of consciousness of unspecified duration, sequela
S06320A	Contusion and laceration of left cerebrum without loss of consciousness, initial encounter
S06320D	Contusion and laceration of left cerebrum without loss of consciousness, subsequent encounter
S06320S	Contusion and laceration of left cerebrum without loss of consciousness, sequela
S06321A	Contusion and laceration of left cerebrum with loss of consciousness of 30 minutes or less, initial encounter
S06321D	Contusion and laceration of left cerebrum with loss of consciousness of 30 minutes or less, subsequent encounter
S06321S	Contusion and laceration of left cerebrum with loss of consciousness of 30 minutes or less, sequela
S06322A	Contusion and laceration of left cerebrum with loss of consciousness of 31 minutes to 59 minutes, initial encounter
S06322D	Contusion and laceration of left cerebrum with loss of consciousness of 31 minutes to 59 minutes, subsequent encounter
S06322S	Contusion and laceration of left cerebrum with loss of consciousness of 31 minutes to 59 minutes, sequela
S06323A	Contusion and laceration of left cerebrum with loss of consciousness of 1 hour to 5 hours 59 minutes, initial encounter
S06323D	Contusion and laceration of left cerebrum with loss of consciousness of 1 hour to 5 hours 59 minutes, subsequent encounter
S06323S	Contusion and laceration of left cerebrum with loss of consciousness of 1 hour to 5 hours 59 minutes, sequela
S06324A	Contusion and laceration of left cerebrum with loss of consciousness of 6 hours to 24 hours, initial encounter
S06324D	Contusion and laceration of left cerebrum with loss of consciousness of 6 hours to 24 hours, subsequent encounter
S06324S	Contusion and laceration of left cerebrum with loss of consciousness of 6 hours to 24 hours, sequela
S06325A	Contusion and laceration of left cerebrum with loss of consciousness greater than 24 hours with return to pre-existing conscious level, initial encounter
S06325D	Contusion and laceration of left cerebrum with loss of consciousness greater than 24 hours with return to pre-existing conscious level, subsequent encounter
S06325S	Contusion and laceration of left cerebrum with loss of consciousness greater than 24 hours with return to pre-existing conscious level, sequela
S06326A	Contusion and laceration of left cerebrum with loss of consciousness greater than 24 hours without return to pre-existing conscious level with patient surviving, initial encounter
S06326D	Contusion and laceration of left cerebrum with loss of consciousness greater than 24 hours without return to pre-existing conscious level with patient surviving, subsequent encounter

S06326S	Contusion and laceration of left cerebrum with loss of consciousness greater than 24 hours without return to pre-existing conscious level with patient surviving, sequela
S06327A	Contusion and laceration of left cerebrum with loss of consciousness of any duration with death due to brain injury prior to regaining consciousness, initial encounter
S06328A	Contusion and laceration of left cerebrum with loss of consciousness of any duration with death due to other cause prior to regaining consciousness, initial encounter
S06329A	Contusion and laceration of left cerebrum with loss of consciousness of unspecified duration, initial encounter
S06329D	Contusion and laceration of left cerebrum with loss of consciousness of unspecified duration, subsequent encounter
S06329S	Contusion and laceration of left cerebrum with loss of consciousness of unspecified duration, sequela
S06330A	Contusion and laceration of cerebrum, unspecified, without loss of consciousness, initial encounter
S06330D	Contusion and laceration of cerebrum, unspecified, without loss of consciousness, subsequent encounter
S06330S	Contusion and laceration of cerebrum, unspecified, without loss of consciousness, sequela
S06331A	Contusion and laceration of cerebrum, unspecified, with loss of consciousness of 30 minutes or less, initial encounter
S06331D	Contusion and laceration of cerebrum, unspecified, with loss of consciousness of 30 minutes or less, subsequent encounter
S06331S	Contusion and laceration of cerebrum, unspecified, with loss of consciousness of 30 minutes or less, sequela
S06332A	Contusion and laceration of cerebrum, unspecified, with loss of consciousness of 31 minutes to 59 minutes, initial encounter
S06332D	Contusion and laceration of cerebrum, unspecified, with loss of consciousness of 31 minutes to 59 minutes, subsequent encounter
S06332S	Contusion and laceration of cerebrum, unspecified, with loss of consciousness of 31 minutes to 59 minutes, sequela
S06333A	Contusion and laceration of cerebrum, unspecified, with loss of consciousness of 1 hour to 5 hours 59 minutes, initial encounter
S06333D	Contusion and laceration of cerebrum, unspecified, with loss of consciousness of 1 hour to 5 hours 59 minutes, subsequent encounter
S06333S	Contusion and laceration of cerebrum, unspecified, with loss of consciousness of 1 hour to 5 hours 59 minutes, sequela
S06334A	Contusion and laceration of cerebrum, unspecified, with loss of consciousness of 6 hours to 24 hours, initial encounter
S06334D	Contusion and laceration of cerebrum, unspecified, with loss of consciousness of 6 hours to 24 hours, subsequent encounter
S06334S	Contusion and laceration of cerebrum, unspecified, with loss of consciousness of 6 hours to 24 hours, sequela
S06335A	Contusion and laceration of cerebrum, unspecified, with loss of consciousness greater than 24 hours with return to pre-existing conscious level, initial encounter
S06335D	Contusion and laceration of cerebrum, unspecified, with loss of consciousness greater than 24 hours with return to pre-existing conscious level, subsequent encounter

S06335S	Contusion and laceration of cerebrum, unspecified, with loss of consciousness greater than 24 hours with return to pre-existing conscious level, sequela
S06336A	Contusion and laceration of cerebrum, unspecified, with loss of consciousness greater than 24 hours without return to pre-existing conscious level with patient surviving, initial encounter
S06336D	Contusion and laceration of cerebrum, unspecified, with loss of consciousness greater than 24 hours without return to pre-existing conscious level with patient surviving, subsequent encounter
S06336S	Contusion and laceration of cerebrum, unspecified, with loss of consciousness greater than 24 hours without return to pre-existing conscious level with patient surviving, sequela
S06337A	Contusion and laceration of cerebrum, unspecified, with loss of consciousness of any duration with death due to brain injury prior to regaining consciousness, initial encounter
S06338A	Contusion and laceration of cerebrum, unspecified, with loss of consciousness of any duration with death due to other cause prior to regaining consciousness, initial encounter
S06339A	Contusion and laceration of cerebrum, unspecified, with loss of consciousness of unspecified duration, initial encounter
S06339D	Contusion and laceration of cerebrum, unspecified, with loss of consciousness of unspecified duration, subsequent encounter
S06339S	Contusion and laceration of cerebrum, unspecified, with loss of consciousness of unspecified duration, sequela
S06340A	Traumatic hemorrhage of right cerebrum without loss of consciousness, initial encounter
S06340D	Traumatic hemorrhage of right cerebrum without loss of consciousness, subsequent encounter
S06340S	Traumatic hemorrhage of right cerebrum without loss of consciousness, sequela
S06341A	Traumatic hemorrhage of right cerebrum with loss of consciousness of 30 minutes or less, initial encounter
S06341D	Traumatic hemorrhage of right cerebrum with loss of consciousness of 30 minutes or less, subsequent encounter
S06341S	Traumatic hemorrhage of right cerebrum with loss of consciousness of 30 minutes or less, sequela
S06342A	Traumatic hemorrhage of right cerebrum with loss of consciousness of 31 minutes to 59 minutes, initial encounter
S06342D	Traumatic hemorrhage of right cerebrum with loss of consciousness of 31 minutes to 59 minutes, subsequent encounter
S06342S	Traumatic hemorrhage of right cerebrum with loss of consciousness of 31 minutes to 59 minutes, sequela
S06343A	Traumatic hemorrhage of right cerebrum with loss of consciousness of 1 hours to 5 hours 59 minutes, initial encounter
S06343D	Traumatic hemorrhage of right cerebrum with loss of consciousness of 1 hours to 5 hours 59 minutes, subsequent encounter
S06343S	Traumatic hemorrhage of right cerebrum with loss of consciousness of 1 hours to 5 hours 59 minutes, sequela
S06344A	Traumatic hemorrhage of right cerebrum with loss of consciousness of 6 hours to 24 hours, initial encounter

S06344D	Traumatic hemorrhage of right cerebrum with loss of consciousness of 6 hours to 24 hours, subsequent encounter
S06344S	Traumatic hemorrhage of right cerebrum with loss of consciousness of 6 hours to 24 hours, sequela
S06345A	Traumatic hemorrhage of right cerebrum with loss of consciousness greater than 24 hours with return to pre-existing conscious level, initial encounter
S06345D	Traumatic hemorrhage of right cerebrum with loss of consciousness greater than 24 hours with return to pre-existing conscious level, subsequent encounter
S06345S	Traumatic hemorrhage of right cerebrum with loss of consciousness greater than 24 hours with return to pre-existing conscious level, sequela
S06346A	Traumatic hemorrhage of right cerebrum with loss of consciousness greater than 24 hours without return to pre-existing conscious level with patient surviving, initial encounter
S06346D	Traumatic hemorrhage of right cerebrum with loss of consciousness greater than 24 hours without return to pre-existing conscious level with patient surviving, subsequent encounter
S06346S	Traumatic hemorrhage of right cerebrum with loss of consciousness greater than 24 hours without return to pre-existing conscious level with patient surviving, sequela
S06347A	Traumatic hemorrhage of right cerebrum with loss of consciousness of any duration with death due to brain injury prior to regaining consciousness, initial encounter
S06348A	Traumatic hemorrhage of right cerebrum with loss of consciousness of any duration with death due to other cause prior to regaining consciousness, initial encounter
S06349A	Traumatic hemorrhage of right cerebrum with loss of consciousness of unspecified duration, initial encounter
S06349D	Traumatic hemorrhage of right cerebrum with loss of consciousness of unspecified duration, subsequent encounter
S06349S	Traumatic hemorrhage of right cerebrum with loss of consciousness of unspecified duration, sequela
S06350A	Traumatic hemorrhage of left cerebrum without loss of consciousness, initial encounter
S06350D	Traumatic hemorrhage of left cerebrum without loss of consciousness, subsequent encounter
S06350S	Traumatic hemorrhage of left cerebrum without loss of consciousness, sequela
S06351A	Traumatic hemorrhage of left cerebrum with loss of consciousness of 30 minutes or less, initial encounter
S06351D	Traumatic hemorrhage of left cerebrum with loss of consciousness of 30 minutes or less, subsequent encounter
S06351S	Traumatic hemorrhage of left cerebrum with loss of consciousness of 30 minutes or less, sequela
S06352A	Traumatic hemorrhage of left cerebrum with loss of consciousness of 31 minutes to 59 minutes, initial encounter
S06352D	Traumatic hemorrhage of left cerebrum with loss of consciousness of 31 minutes to 59 minutes, subsequent encounter
S06352S	Traumatic hemorrhage of left cerebrum with loss of consciousness of 31 minutes to 59 minutes, sequela
S06353A	Traumatic hemorrhage of left cerebrum with loss of consciousness of 1 hours to 5 hours 59 minutes, initial encounter

S06353D	Traumatic hemorrhage of left cerebrum with loss of consciousness of 1 hours to 5 hours 59 minutes, subsequent encounter
S06353S	Traumatic hemorrhage of left cerebrum with loss of consciousness of 1 hours to 5 hours 59 minutes, sequela
S06354A	Traumatic hemorrhage of left cerebrum with loss of consciousness of 6 hours to 24 hours, initial encounter
S06354D	Traumatic hemorrhage of left cerebrum with loss of consciousness of 6 hours to 24 hours, subsequent encounter
S06354S	Traumatic hemorrhage of left cerebrum with loss of consciousness of 6 hours to 24 hours, sequela
S06355A	Traumatic hemorrhage of left cerebrum with loss of consciousness greater than 24 hours with return to pre-existing conscious level, initial encounter
S06355D	Traumatic hemorrhage of left cerebrum with loss of consciousness greater than 24 hours with return to pre-existing conscious level, subsequent encounter
S06355S	Traumatic hemorrhage of left cerebrum with loss of consciousness greater than 24 hours with return to pre-existing conscious level, sequela
S06356A	Traumatic hemorrhage of left cerebrum with loss of consciousness greater than 24 hours without return to pre-existing conscious level with patient surviving, initial encounter
S06356D	Traumatic hemorrhage of left cerebrum with loss of consciousness greater than 24 hours without return to pre-existing conscious level with patient surviving, subsequent encounter
S06356S	Traumatic hemorrhage of left cerebrum with loss of consciousness greater than 24 hours without return to pre-existing conscious level with patient surviving, sequela
S06357A	Traumatic hemorrhage of left cerebrum with loss of consciousness of any duration with death due to brain injury prior to regaining consciousness, initial encounter
S06358A	Traumatic hemorrhage of left cerebrum with loss of consciousness of any duration with death due to other cause prior to regaining consciousness, initial encounter
S06359A	Traumatic hemorrhage of left cerebrum with loss of consciousness of unspecified duration, initial encounter
S06359D	Traumatic hemorrhage of left cerebrum with loss of consciousness of unspecified duration, subsequent encounter
S06359S	Traumatic hemorrhage of left cerebrum with loss of consciousness of unspecified duration, sequela
S06360A	Traumatic hemorrhage of cerebrum, unspecified, without loss of consciousness, initial encounter
S06360D	Traumatic hemorrhage of cerebrum, unspecified, without loss of consciousness, subsequent encounter
S06360S	Traumatic hemorrhage of cerebrum, unspecified, without loss of consciousness, sequela
S06361A	Traumatic hemorrhage of cerebrum, unspecified, with loss of consciousness of 30 minutes or less, initial encounter
S06361D	Traumatic hemorrhage of cerebrum, unspecified, with loss of consciousness of 30 minutes or less, subsequent encounter
S06361S	Traumatic hemorrhage of cerebrum, unspecified, with loss of consciousness of 30 minutes or less, sequela
S06362A	Traumatic hemorrhage of cerebrum, unspecified, with loss of consciousness of 31 minutes to 59 minutes, initial encounter

S06362D	Traumatic hemorrhage of cerebrum, unspecified, with loss of consciousness of 31 minutes to 59 minutes, subsequent encounter
S06362S	Traumatic hemorrhage of cerebrum, unspecified, with loss of consciousness of 31 minutes to 59 minutes, sequela
S06363A	Traumatic hemorrhage of cerebrum, unspecified, with loss of consciousness of 1 hours to 5 hours 59 minutes, initial encounter
S06363D	Traumatic hemorrhage of cerebrum, unspecified, with loss of consciousness of 1 hours to 5 hours 59 minutes, subsequent encounter
S06363S	Traumatic hemorrhage of cerebrum, unspecified, with loss of consciousness of 1 hours to 5 hours 59 minutes, sequela
S06364A	Traumatic hemorrhage of cerebrum, unspecified, with loss of consciousness of 6 hours to 24 hours, initial encounter
S06364D	Traumatic hemorrhage of cerebrum, unspecified, with loss of consciousness of 6 hours to 24 hours, subsequent encounter
S06364S	Traumatic hemorrhage of cerebrum, unspecified, with loss of consciousness of 6 hours to 24 hours, sequela
S06365A	Traumatic hemorrhage of cerebrum, unspecified, with loss of consciousness greater than 24 hours with return to pre-existing conscious level, initial encounter
S06365D	Traumatic hemorrhage of cerebrum, unspecified, with loss of consciousness greater than 24 hours with return to pre-existing conscious level, subsequent encounter
S06365S	Traumatic hemorrhage of cerebrum, unspecified, with loss of consciousness greater than 24 hours with return to pre-existing conscious level, sequela
S06366A	Traumatic hemorrhage of cerebrum, unspecified, with loss of consciousness greater than 24 hours without return to pre-existing conscious level with patient surviving, initial encounter
S06366D	Traumatic hemorrhage of cerebrum, unspecified, with loss of consciousness greater than 24 hours without return to pre-existing conscious level with patient surviving, subsequent encounter
S06366S	Traumatic hemorrhage of cerebrum, unspecified, with loss of consciousness greater than 24 hours without return to pre-existing conscious level with patient surviving, sequela
S06367A	Traumatic hemorrhage of cerebrum, unspecified, with loss of consciousness of any duration with death due to brain injury prior to regaining consciousness, initial encounter
S06368A	Traumatic hemorrhage of cerebrum, unspecified, with loss of consciousness of any duration with death due to other cause prior to regaining consciousness, initial encounter
S06369A	Traumatic hemorrhage of cerebrum, unspecified, with loss of consciousness of unspecified duration, initial encounter
S06369D	Traumatic hemorrhage of cerebrum, unspecified, with loss of consciousness of unspecified duration, subsequent encounter
S06369S	Traumatic hemorrhage of cerebrum, unspecified, with loss of consciousness of unspecified duration, sequela
S06370A	Contusion, laceration, and hemorrhage of cerebellum without loss of consciousness, initial encounter
S06370D	Contusion, laceration, and hemorrhage of cerebellum without loss of consciousness, subsequent encounter
S06370S	Contusion, laceration, and hemorrhage of cerebellum without loss of consciousness, sequela

S06371A	Contusion, laceration, and hemorrhage of cerebellum with loss of consciousness of 30 minutes or less, initial encounter
S06371D	Contusion, laceration, and hemorrhage of cerebellum with loss of consciousness of 30 minutes or less, subsequent encounter
S06371S	Contusion, laceration, and hemorrhage of cerebellum with loss of consciousness of 30 minutes or less, sequela
S06372A	Contusion, laceration, and hemorrhage of cerebellum with loss of consciousness of 31 minutes to 59 minutes, initial encounter
S06372D	Contusion, laceration, and hemorrhage of cerebellum with loss of consciousness of 31 minutes to 59 minutes, subsequent encounter
S06372S	Contusion, laceration, and hemorrhage of cerebellum with loss of consciousness of 31 minutes to 59 minutes, sequela
S06373A	Contusion, laceration, and hemorrhage of cerebellum with loss of consciousness of 1 hour to 5 hours 59 minutes, initial encounter
S06373D	Contusion, laceration, and hemorrhage of cerebellum with loss of consciousness of 1 hour to 5 hours 59 minutes, subsequent encounter
S06373S	Contusion, laceration, and hemorrhage of cerebellum with loss of consciousness of 1 hour to 5 hours 59 minutes, sequela
S06374A	Contusion, laceration, and hemorrhage of cerebellum with loss of consciousness of 6 hours to 24 hours, initial encounter
S06374D	Contusion, laceration, and hemorrhage of cerebellum with loss of consciousness of 6 hours to 24 hours, subsequent encounter
S06374S	Contusion, laceration, and hemorrhage of cerebellum with loss of consciousness of 6 hours to 24 hours, sequela
S06375A	Contusion, laceration, and hemorrhage of cerebellum with loss of consciousness greater than 24 hours with return to pre-existing conscious level, initial encounter
S06375D	Contusion, laceration, and hemorrhage of cerebellum with loss of consciousness greater than 24 hours with return to pre-existing conscious level, subsequent encounter
S06375S	Contusion, laceration, and hemorrhage of cerebellum with loss of consciousness greater than 24 hours with return to pre-existing conscious level, sequela
S06376A	Contusion, laceration, and hemorrhage of cerebellum with loss of consciousness greater than 24 hours without return to pre-existing conscious level with patient surviving, initial encounter
S06376D	Contusion, laceration, and hemorrhage of cerebellum with loss of consciousness greater than 24 hours without return to pre-existing conscious level with patient surviving, subsequent encounter
S06376S	Contusion, laceration, and hemorrhage of cerebellum with loss of consciousness greater than 24 hours without return to pre-existing conscious level with patient surviving, sequela
S06377A	Contusion, laceration, and hemorrhage of cerebellum with loss of consciousness of any duration with death due to brain injury prior to regaining consciousness, initial encounter
S06378A	Contusion, laceration, and hemorrhage of cerebellum with loss of consciousness of any duration with death due to other cause prior to regaining consciousness, initial encounter
S06379A	Contusion, laceration, and hemorrhage of cerebellum with loss of consciousness of unspecified duration, initial encounter
S06379D	Contusion, laceration, and hemorrhage of cerebellum with loss of consciousness of unspecified duration, subsequent encounter

S06379S	Contusion, laceration, and hemorrhage of cerebellum with loss of consciousness of unspecified duration, sequela
S06380A	Contusion, laceration, and hemorrhage of brainstem without loss of consciousness, initial encounter
S06380D	Contusion, laceration, and hemorrhage of brainstem without loss of consciousness, subsequent encounter
S06380S	Contusion, laceration, and hemorrhage of brainstem without loss of consciousness, sequela
S06381A	Contusion, laceration, and hemorrhage of brainstem with loss of consciousness of 30 minutes or less, initial encounter
S06381D	Contusion, laceration, and hemorrhage of brainstem with loss of consciousness of 30 minutes or less, subsequent encounter
S06381S	Contusion, laceration, and hemorrhage of brainstem with loss of consciousness of 30 minutes or less, sequela
S06382A	Contusion, laceration, and hemorrhage of brainstem with loss of consciousness of 31 minutes to 59 minutes, initial encounter
S06382D	Contusion, laceration, and hemorrhage of brainstem with loss of consciousness of 31 minutes to 59 minutes, subsequent encounter
S06382S	Contusion, laceration, and hemorrhage of brainstem with loss of consciousness of 31 minutes to 59 minutes, sequela
S06383A	Contusion, laceration, and hemorrhage of brainstem with loss of consciousness of 1 hour to 5 hours 59 minutes, initial encounter
S06383D	Contusion, laceration, and hemorrhage of brainstem with loss of consciousness of 1 hour to 5 hours 59 minutes, subsequent encounter
S06383S	Contusion, laceration, and hemorrhage of brainstem with loss of consciousness of 1 hour to 5 hours 59 minutes, sequela
S06384A	Contusion, laceration, and hemorrhage of brainstem with loss of consciousness of 6 hours to 24 hours, initial encounter
S06384D	Contusion, laceration, and hemorrhage of brainstem with loss of consciousness of 6 hours to 24 hours, subsequent encounter
S06384S	Contusion, laceration, and hemorrhage of brainstem with loss of consciousness of 6 hours to 24 hours, sequela
S06385A	Contusion, laceration, and hemorrhage of brainstem with loss of consciousness greater than 24 hours with return to pre-existing conscious level, initial encounter
S06385D	Contusion, laceration, and hemorrhage of brainstem with loss of consciousness greater than 24 hours with return to pre-existing conscious level, subsequent encounter
S06385S	Contusion, laceration, and hemorrhage of brainstem with loss of consciousness greater than 24 hours with return to pre-existing conscious level, sequela
S06386A	Contusion, laceration, and hemorrhage of brainstem with loss of consciousness greater than 24 hours without return to pre-existing conscious level with patient surviving, initial encounter
S06386D	Contusion, laceration, and hemorrhage of brainstem with loss of consciousness greater than 24 hours without return to pre-existing conscious level with patient surviving, subsequent encounter
S06386S	Contusion, laceration, and hemorrhage of brainstem with loss of consciousness greater than 24 hours without return to pre-existing conscious level with patient surviving, sequela

S06387A	Contusion, laceration, and hemorrhage of brainstem with loss of consciousness of any duration with death due to brain injury prior to regaining consciousness, initial encounter
S06388A	Contusion, laceration, and hemorrhage of brainstem with loss of consciousness of any duration with death due to other cause prior to regaining consciousness, initial encounter
S06389A	Contusion, laceration, and hemorrhage of brainstem with loss of consciousness of unspecified duration, initial encounter
S06389D	Contusion, laceration, and hemorrhage of brainstem with loss of consciousness of unspecified duration, subsequent encounter
S06389S	Contusion, laceration, and hemorrhage of brainstem with loss of consciousness of unspecified duration, sequela
S064X0A	Epidural hemorrhage without loss of consciousness, initial encounter
S064X0D	Epidural hemorrhage without loss of consciousness, subsequent encounter
S064X0S	Epidural hemorrhage without loss of consciousness, sequela
S064X1A	Epidural hemorrhage with loss of consciousness of 30 minutes or less, initial encounter
S064X1D	Epidural hemorrhage with loss of consciousness of 30 minutes or less, subsequent encounter
S064X1S	Epidural hemorrhage with loss of consciousness of 30 minutes or less, sequela
S064X2A	Epidural hemorrhage with loss of consciousness of 31 minutes to 59 minutes, initial encounter
S064X2D	Epidural hemorrhage with loss of consciousness of 31 minutes to 59 minutes, subsequent encounter
S064X2S	Epidural hemorrhage with loss of consciousness of 31 minutes to 59 minutes, sequela
S064X3A	Epidural hemorrhage with loss of consciousness of 1 hour to 5 hours 59 minutes, initial encounter
S064X3D	Epidural hemorrhage with loss of consciousness of 1 hour to 5 hours 59 minutes, subsequent encounter
S064X3S	Epidural hemorrhage with loss of consciousness of 1 hour to 5 hours 59 minutes, sequela
S064X4A	Epidural hemorrhage with loss of consciousness of 6 hours to 24 hours, initial encounter
S064X4D	Epidural hemorrhage with loss of consciousness of 6 hours to 24 hours, subsequent encounter
S064X4S	Epidural hemorrhage with loss of consciousness of 6 hours to 24 hours, sequela
S064X5A	Epidural hemorrhage with loss of consciousness greater than 24 hours with return to pre-existing conscious level, initial encounter
S064X5D	Epidural hemorrhage with loss of consciousness greater than 24 hours with return to pre-existing conscious level, subsequent encounter
S064X5S	Epidural hemorrhage with loss of consciousness greater than 24 hours with return to pre-existing conscious level, sequela
S064X6A	Epidural hemorrhage with loss of consciousness greater than 24 hours without return to pre-existing conscious level with patient surviving, initial encounter
S064X6D	Epidural hemorrhage with loss of consciousness greater than 24 hours without return to pre-existing conscious level with patient surviving, subsequent encounter
S064X6S	Epidural hemorrhage with loss of consciousness greater than 24 hours without return to pre-existing conscious level with patient surviving, sequela

S064X7A	Epidural hemorrhage with loss of consciousness of any duration with death due to brain injury prior to regaining consciousness, initial encounter
S064X8A	Epidural hemorrhage with loss of consciousness of any duration with death due to other causes prior to regaining consciousness, initial encounter
S064X9A	Epidural hemorrhage with loss of consciousness of unspecified duration, initial encounter
S064X9D	Epidural hemorrhage with loss of consciousness of unspecified duration, subsequent encounter
S064X9S	Epidural hemorrhage with loss of consciousness of unspecified duration, sequela
S065X0A	Traumatic subdural hemorrhage without loss of consciousness, initial encounter
S065X0D	Traumatic subdural hemorrhage without loss of consciousness, subsequent encounter
S065X0S	Traumatic subdural hemorrhage without loss of consciousness, sequela
S065X1A	Traumatic subdural hemorrhage with loss of consciousness of 30 minutes or less, initial encounter
S065X1D	Traumatic subdural hemorrhage with loss of consciousness of 30 minutes or less, subsequent encounter
S065X1S	Traumatic subdural hemorrhage with loss of consciousness of 30 minutes or less, sequela
S065X2A	Traumatic subdural hemorrhage with loss of consciousness of 31 minutes to 59 minutes, initial encounter
S065X2D	Traumatic subdural hemorrhage with loss of consciousness of 31 minutes to 59 minutes, subsequent encounter
S065X2S	Traumatic subdural hemorrhage with loss of consciousness of 31 minutes to 59 minutes, sequela
S065X3A	Traumatic subdural hemorrhage with loss of consciousness of 1 hour to 5 hours 59 minutes, initial encounter
S065X3D	Traumatic subdural hemorrhage with loss of consciousness of 1 hour to 5 hours 59 minutes, subsequent encounter
S065X3S	Traumatic subdural hemorrhage with loss of consciousness of 1 hour to 5 hours 59 minutes, sequela
S065X4A	Traumatic subdural hemorrhage with loss of consciousness of 6 hours to 24 hours, initial encounter
S065X4D	Traumatic subdural hemorrhage with loss of consciousness of 6 hours to 24 hours, subsequent encounter
S065X4S	Traumatic subdural hemorrhage with loss of consciousness of 6 hours to 24 hours, sequela
S065X5A	Traumatic subdural hemorrhage with loss of consciousness greater than 24 hours with return to pre-existing conscious level, initial encounter
S065X5D	Traumatic subdural hemorrhage with loss of consciousness greater than 24 hours with return to pre-existing conscious level, subsequent encounter
S065X5S	Traumatic subdural hemorrhage with loss of consciousness greater than 24 hours with return to pre-existing conscious level, sequela
S065X6A	Traumatic subdural hemorrhage with loss of consciousness greater than 24 hours without return to pre-existing conscious level with patient surviving, initial encounter
S065X6D	Traumatic subdural hemorrhage with loss of consciousness greater than 24 hours without return to pre-existing conscious level with patient surviving, subsequent encounter
S065X6S	Traumatic subdural hemorrhage with loss of consciousness greater than 24 hours without return to pre-existing conscious level with patient surviving, sequela

S065X7A	Traumatic subdural hemorrhage with loss of consciousness of any duration with death due to brain injury before regaining consciousness, initial encounter
S065X8A	Traumatic subdural hemorrhage with loss of consciousness of any duration with death due to other cause before regaining consciousness, initial encounter
S065X9A	Traumatic subdural hemorrhage with loss of consciousness of unspecified duration, initial encounter
S065X9D	Traumatic subdural hemorrhage with loss of consciousness of unspecified duration, subsequent encounter
S065X9S	Traumatic subdural hemorrhage with loss of consciousness of unspecified duration, sequela
S066X0A	Traumatic subarachnoid hemorrhage without loss of consciousness, initial encounter
S066X0D	Traumatic subarachnoid hemorrhage without loss of consciousness, subsequent encounter
S066X0S	Traumatic subarachnoid hemorrhage without loss of consciousness, sequela
S066X1A	Traumatic subarachnoid hemorrhage with loss of consciousness of 30 minutes or less, initial encounter
S066X1D	Traumatic subarachnoid hemorrhage with loss of consciousness of 30 minutes or less, subsequent encounter
S066X1S	Traumatic subarachnoid hemorrhage with loss of consciousness of 30 minutes or less, sequela
S066X2A	Traumatic subarachnoid hemorrhage with loss of consciousness of 31 minutes to 59 minutes, initial encounter
S066X2D	Traumatic subarachnoid hemorrhage with loss of consciousness of 31 minutes to 59 minutes, subsequent encounter
S066X2S	Traumatic subarachnoid hemorrhage with loss of consciousness of 31 minutes to 59 minutes, sequela
S066X3A	Traumatic subarachnoid hemorrhage with loss of consciousness of 1 hour to 5 hours 59 minutes, initial encounter
S066X3D	Traumatic subarachnoid hemorrhage with loss of consciousness of 1 hour to 5 hours 59 minutes, subsequent encounter
S066X3S	Traumatic subarachnoid hemorrhage with loss of consciousness of 1 hour to 5 hours 59 minutes, sequela
S066X4A	Traumatic subarachnoid hemorrhage with loss of consciousness of 6 hours to 24 hours, initial encounter
S066X4D	Traumatic subarachnoid hemorrhage with loss of consciousness of 6 hours to 24 hours, subsequent encounter
S066X4S	Traumatic subarachnoid hemorrhage with loss of consciousness of 6 hours to 24 hours, sequela
S066X5A	Traumatic subarachnoid hemorrhage with loss of consciousness greater than 24 hours with return to pre-existing conscious level, initial encounter
S066X5D	Traumatic subarachnoid hemorrhage with loss of consciousness greater than 24 hours with return to pre-existing conscious level, subsequent encounter
S066X5S	Traumatic subarachnoid hemorrhage with loss of consciousness greater than 24 hours with return to pre-existing conscious level, sequela
S066X6A	Traumatic subarachnoid hemorrhage with loss of consciousness greater than 24 hours without return to pre-existing conscious level with patient surviving, initial encounter

S066X6D	Traumatic subarachnoid hemorrhage with loss of consciousness greater than 24 hours without return to pre-existing conscious level with patient surviving, subsequent encounter
S066X6S	Traumatic subarachnoid hemorrhage with loss of consciousness greater than 24 hours without return to pre-existing conscious level with patient surviving, sequela
S066X7A	Traumatic subarachnoid hemorrhage with loss of consciousness of any duration with death due to brain injury prior to regaining consciousness, initial encounter
S066X8A	Traumatic subarachnoid hemorrhage with loss of consciousness of any duration with death due to other cause prior to regaining consciousness, initial encounter
S066X9A	Traumatic subarachnoid hemorrhage with loss of consciousness of unspecified duration, initial encounter
S066X9D	Traumatic subarachnoid hemorrhage with loss of consciousness of unspecified duration, subsequent encounter
S066X9S	Traumatic subarachnoid hemorrhage with loss of consciousness of unspecified duration, sequela
S06890A	Other specified intracranial injury without loss of consciousness, initial encounter
S06890D	Other specified intracranial injury without loss of consciousness, subsequent encounter
S06890S	Other specified intracranial injury without loss of consciousness, sequela
S06891A	Other specified intracranial injury with loss of consciousness of 30 minutes or less, initial encounter
S06891D	Other specified intracranial injury with loss of consciousness of 30 minutes or less, subsequent encounter
S06891S	Other specified intracranial injury with loss of consciousness of 30 minutes or less, sequela
S06892A	Other specified intracranial injury with loss of consciousness of 31 minutes to 59 minutes, initial encounter
S06892D	Other specified intracranial injury with loss of consciousness of 31 minutes to 59 minutes, subsequent encounter
S06892S	Other specified intracranial injury with loss of consciousness of 31 minutes to 59 minutes, sequela
S06893A	Other specified intracranial injury with loss of consciousness of 1 hour to 5 hours 59 minutes, initial encounter
S06893D	Other specified intracranial injury with loss of consciousness of 1 hour to 5 hours 59 minutes, subsequent encounter
S06893S	Other specified intracranial injury with loss of consciousness of 1 hour to 5 hours 59 minutes, sequela
S06894A	Other specified intracranial injury with loss of consciousness of 6 hours to 24 hours, initial encounter
S06894D	Other specified intracranial injury with loss of consciousness of 6 hours to 24 hours, subsequent encounter
S06894S	Other specified intracranial injury with loss of consciousness of 6 hours to 24 hours, sequela
S06895A	Other specified intracranial injury with loss of consciousness greater than 24 hours with return to pre-existing conscious level, initial encounter
S06895D	Other specified intracranial injury with loss of consciousness greater than 24 hours with return to pre-existing conscious level, subsequent encounter

S06895S	Other specified intracranial injury with loss of consciousness greater than 24 hours with return to pre-existing conscious level, sequela
S06896A	Other specified intracranial injury with loss of consciousness greater than 24 hours without return to pre-existing conscious level with patient surviving, initial encounter
S06896D	Other specified intracranial injury with loss of consciousness greater than 24 hours without return to pre-existing conscious level with patient surviving, subsequent encounter
S06896S	Other specified intracranial injury with loss of consciousness greater than 24 hours without return to pre-existing conscious level with patient surviving, sequela
S06897A	Other specified intracranial injury with loss of consciousness of any duration with death due to brain injury prior to regaining consciousness, initial encounter
S06898A	Other specified intracranial injury with loss of consciousness of any duration with death due to other cause prior to regaining consciousness, initial encounter
S06899A	Other specified intracranial injury with loss of consciousness of unspecified duration, initial encounter
S06899D	Other specified intracranial injury with loss of consciousness of unspecified duration, subsequent encounter
S06899S	Other specified intracranial injury with loss of consciousness of unspecified duration, sequela
S069X0A	Unspecified intracranial injury without loss of consciousness, initial encounter
S069X0D	Unspecified intracranial injury without loss of consciousness, subsequent encounter
S069X0S	Unspecified intracranial injury without loss of consciousness, sequela
S069X1A	Unspecified intracranial injury with loss of consciousness of 30 minutes or less, initial encounter
S069X1D	Unspecified intracranial injury with loss of consciousness of 30 minutes or less, subsequent encounter
S069X1S	Unspecified intracranial injury with loss of consciousness of 30 minutes or less, sequela
S069X2A	Unspecified intracranial injury with loss of consciousness of 31 minutes to 59 minutes, initial encounter
S069X2D	Unspecified intracranial injury with loss of consciousness of 31 minutes to 59 minutes, subsequent encounter
S069X2S	Unspecified intracranial injury with loss of consciousness of 31 minutes to 59 minutes, sequela
S069X3A	Unspecified intracranial injury with loss of consciousness of 1 hour to 5 hours 59 minutes, initial encounter
S069X3D	Unspecified intracranial injury with loss of consciousness of 1 hour to 5 hours 59 minutes, subsequent encounter
S069X3S	Unspecified intracranial injury with loss of consciousness of 1 hour to 5 hours 59 minutes, sequela
S069X4A	Unspecified intracranial injury with loss of consciousness of 6 hours to 24 hours, initial encounter
S069X4D	Unspecified intracranial injury with loss of consciousness of 6 hours to 24 hours, subsequent encounter
S069X4S	Unspecified intracranial injury with loss of consciousness of 6 hours to 24 hours, sequela
S069X5A	Unspecified intracranial injury with loss of consciousness greater than 24 hours with return to pre-existing conscious level, initial encounter

S069X5D	Unspecified intracranial injury with loss of consciousness greater than 24 hours with return to pre-existing conscious level, subsequent encounter
S069X5S	Unspecified intracranial injury with loss of consciousness greater than 24 hours with return to pre-existing conscious level, sequela
S069X6A	Unspecified intracranial injury with loss of consciousness greater than 24 hours without return to pre-existing conscious level with patient surviving, initial encounter
S069X6D	Unspecified intracranial injury with loss of consciousness greater than 24 hours without return to pre-existing conscious level with patient surviving, subsequent encounter
S069X6S	Unspecified intracranial injury with loss of consciousness greater than 24 hours without return to pre-existing conscious level with patient surviving, sequela
S069X7A	Unspecified intracranial injury with loss of consciousness of any duration with death due to brain injury prior to regaining consciousness, initial encounter
S069X8A	Unspecified intracranial injury with loss of consciousness of any duration with death due to other cause prior to regaining consciousness, initial encounter
S069X9A	Unspecified intracranial injury with loss of consciousness of unspecified duration, initial encounter
S069X9D	Unspecified intracranial injury with loss of consciousness of unspecified duration, subsequent encounter
S069X9S	Unspecified intracranial injury with loss of consciousness of unspecified duration, sequela

Supplementary Table S2. The categories of sleep-wake disturbances (SWD) after TBI. Sample sizes are provided in parentheses.

Type of SWD	Males	Females	Total
**circadian rhythm sleep disorder**	5.5% (6)	3.1% (3)	4.4% (9)
**difficulty falling asleep at night until the early morning hours[1]**	1.8% (2)	1.0% (1)	1.5% (3)
**difficulty sleeping[1]**	1.8% (2)	1.0% (1)	1.5% (3)
**disturbance in sleep behavior[1]**	5.5% (6)	4.1% (4)	4.8% (10)
**excessive daytime sleepiness[2]**	0.9% (1)	2.0% (2)	1.5% (3)
**frequent nocturnal awakening[1]**	0% (0)	3.1% (3)	1.5% (3)
**hypersomnia[2]**	5.5% (6)	7.1% (7)	6.3% (13)
**insomnia**	22.9% (25)	28.6% (28)	25.6% (53)
**obstructive sleep apnea (OSA)**	10.1% (11)	5.1% (5)	7.7% (16)
**sleep difficulties/sleep disorders[1]**	29.4% (32)	31.6% (31)	30.4% (63)
**sleep patter disturbance[1]**	1.8% (1)	1.0% (1)	1.0% (2)
**other**	15.6% (17)	12.2% (12)	14.0% (29)

[1]Categories combined into "sleep difficulties/sleep disorders"

[2]Categories combined into "hypersomnia/excessive daytime sleepiness"
